# Locus coeruleus activation transforms cortical taste representations

**DOI:** 10.1016/j.celrep.2026.117990

**Published:** 2026-09-15

**Authors:** Will Fan, Paula To, Natale R. Sciolino

**Affiliations:** 1 Department of Physiology and Neurobiology, Storrs, CT 06269, USA; 2 Institute for the Brain and Cognitive Sciences, Storrs, CT 06269, USA; 3 Center for Addiction Sciences, Storrs, CT 06269, USA; 4 Institute for Systems Genomics, Storrs, CT 06269, USA; 5 Department of Biomedical Engineering, Storrs, CT 06269, USA; 6 Department of Psychological Sciences, University of Connecticut, Storrs, CT 06269, USA; 7 Lead contact

## Abstract

Noradrenergic neurons in the locus coeruleus (LC) shape sensory processing, yet how LC activity influences population taste coding remains unclear. Using optogenetic LC activation with miniscope imaging in the gustatory cortex (GC), we examined LC modulation of multiple taste attributes. Phasic LC activation strengthens correlations between neuronal responses and palatability and expands the dynamic range of stimulus representations along a palatability axis. This expansion is driven by an aversive shift in the representations of all tastants except sucrose, the most palatable stimulus. For mixture ratio and concentration, phasic activation expands and rotates attribute axes, potentially reflecting dependencies between these attributes and palatability. These transformations likely arise from multiplicative gain modulation and more flexible tuning changes. Tonic LC activation affects fewer neurons and does not expand attribute axes. Together, the findings show that LC activation reorganizes GC population geometry in a pattern-dependent manner, linking neuromodulation with feeding behavior and affective processing.

## In brief

Fan et al. showed that phasic, but not tonic, locus coeruleus activation strengthens the relationship between cortical activity and palatability while reorganizing taste population geometry to increase separation between highly palatable and less palatable tastes. The findings reveal how neuromodulation rapidly reshapes cortical codes to emphasize behaviorally relevant information.

## Graphical Abstract

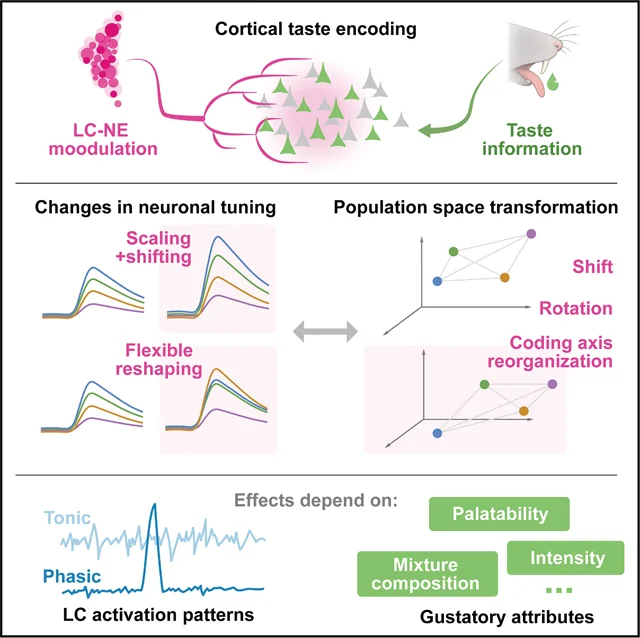

## INTRODUCTION

Sensory processing is adaptively shaped by an animal’s internal state, including fluctuations in arousal, attention, motivation, and stress.^1–6^ One of the best studied sources of modulatory input to sensory circuits is the noradrenergic locus coeruleus (LC).^7–10^ LC activity tracks behavioral state and is commonly described in phasic and tonic firing modes^11^: phasic activation is linked to vigilance, attentional shifts, and motivated actions,^12–14^ whereas elevated tonic firing predominates during stress and hyperarousal.^15,16^ Through its widespread projections to nearly all sensory networks,^17^ the LC can broadly influence circuit dynamics through norepinephrine (NE) release. LC stimulation and pharmacological NE application produce diverse effects across sensory regions, including bidirectional modulation of responses,^18–20^ changes in tuning selectivity,^21–23^ improvement in signal-to-noise ratio,^24,25^ and alterations in response timing.^18,19,26^ These effects depend on brain region^18,27^ and LC activity pattern.^26,28^

Although LC’s modulatory effects have been well-characterized at the single neuron level, it remains unclear how LC activity causally reshapes neural population codes, the level at which perception and behavior emerge.^29,30^ Population responses can be represented as points in high-dimensional activity space, where each axis corresponds to the activity of one neuron. Across stimuli, these points form a low-dimensional manifold whose geometry captures the relationships among stimuli as encoded by the population.^31^ Neuromodulation can scale, shift, rotate, or warp this geometry, with distinct implications for feature encoding and downstream readout. Despite the promise of this approach, only a few studies have applied population-based analyses to LC modulation,^27,28,32^ and how LC activity transforms sensory coding geometry remains poorly understood. Defining these transformations could reveal general principles of how neuromodulators bias sensory processing toward behaviorally relevant stimulus features.

In the present study, we investigated how distinct patterns of LC activation influence taste representations in the primary gustatory cortex (GC) at both the single-neuron and population levels. GC is a strong model system for several reasons. First, compared to other sensory modalities, gustation remains underrepresented in the neuromodulation literature, with no prior work directly testing the causal impact of LC activity on GC coding. Second, GC population activity encodes not only taste identity and intensity, but also *palatability*^33–36^—a behaviorally relevant dimension linking sensory coding to affective processing and feeding behavior. Third, GC palatability coding is flexibly regulated by behavioral states such as attention^37^ and sickness,^38^ but the pathways mediating these effects remain unknown. Given LC’s role in adaptive sensory-guided decisions,^27,39–41^ we hypothesized that LC activation would reorganize GC population geometry by enhancing palatability representations, among other impacts.

To test this hypothesis, we combined miniscope calcium imaging in GC with optogenetic LC activation in awake mice receiving passively delivered taste stimuli. We examined phasic and tonic LC activation across three taste attributes: palatability, mixture ratio, and concentration. Phasic LC activation enhanced the correlation between neuronal responses and palatability and expanded the dynamic range of taste representations along a palatability-relevant axis. Similar stretching occurred for mixture ratio and intensity; however, the corresponding population axes also rotated, potentially due to their correlation with palatability. These population-level changes likely arise from a combination of gain modulation and flexible tuning changes. In contrast, tonic LC stimulation modulated fewer GC neurons and did not produce stretching along any attribute axis, despite inducing greater NE release. Together, these findings show that LC activity reshapes GC population coding in a pattern-dependent manner and provide causal insights into how neuromodulation reorganizes sensory representations of behaviorally relevant stimulus attributes.

## RESULTS

### Probing the effects of phasic LC activation on taste-evoked GC activity

To determine how phasic LC activation influences taste-evoked responses in GC, we expressed GCaMP8m (CaMKIIα-driven) in GC excitatory neurons and the excitatory opsin ChrimsonR (Flp dependent) in NE-producing LC neurons of *Dbh*^Flpo^ mice^17^ (Figure 1A). A relay lens was implanted over GC for miniscope imaging, and an optical fiber was positioned above the ipsilateral LC for optogenetic stimulation. ChrimsonR-expressing LC axons densely innervated GC and intermingled with GCaMP8m-expressing neurons (Figures 1B and 1C).

To control stimulus delivery, mice were implanted with an intraoral cannula (IOC) (Figure 1D). Tastants were delivered through four independent microtubes within the IOC, preventing cross-stimulus mixing. Each session consisted of 96 trials with repeated presentations of four stimuli (Figure 1D). On half of the trials, tastant delivery was preceded by a 0.5-s phasic LC stimulation train (0.5 s; 20 Hz, 5-ms pulses), ending 0.5 s before taste delivery. This stimulation timing to mimic LC activation that precedes feeding^42^ and may prime taste processing. To test whether LC-dependent modulation varied across sensory dimensions, we examined three stimulus sets that differed along defined attributes: palatability (sucrose > NaCl > citric acid > quinine), mixture composition (four sucrose-NaCl ratios), and intensity (four sucrose concentrations) (Figure 1E).

To confirm that this stimulation protocol increased LC output to GC, we performed validation experiments in separate *Dbh*^Flpo^ mice expressing the NE sensor GRAB_NE2h_^43^ in GC and Flp-dependent ChrimsonR in the ipsilateral LC (Figures 1F and 1G). Using spectrally resolved fiber photometry,^42,44,45^ we measured NE dynamics in GC with the same stimulation timing as in miniscope experiments, but without tastant delivery (Figure 1D). Phasic LC stimulation robustly increased GC NE release across mice (Figures 1H and 1I). Although NE fluorescence did not fully return to baseline within the 25–35 s inter-trial interval, this prolonged decay was likely exaggerated by GRAB_NE2h_ off-kinetics (*τ*_off_ = 1.93 s^43^), as NE transients evoked by brief LC or NE pathway stimulation typically return to baseline within 10 s in fast-scan cyclic voltammetry studies.^46–50^ Thus, it is unlikely that endogenous NE signals persist across successive trials. Notably, NE fluorescence signals during stimulation (stim) trials were clearly distinguishable from the no-stimulation (no-stim) trials (Figure 1J), confirming the efficacy of our stimulation protocol and supporting comparisons of GC activity across conditions.

### PHASIC LC ACTIVATION BIDIRECTIONALLY MODULATES TASTE-EVOKED GC RESPONSES

We first examined the effects of phasic LC activation on GC responses to four basic taste stimuli that vary in palatability. We identified neurons exhibiting tastant-dependent responses in stim and/or no-stim trials (hereafter termed *taste-discriminative* neurons). Among these, 20.8% showed differential responses in stim versus no-stim trials for at least one tastant; these were classified as *LC-modulated* neurons (Figure S1A). In tdTomato control mice, only 2.88% of taste-discriminative neurons met this criterion (Figure S1A), suggesting that the effect largely reflect LC activation rather than photo-stimulation artifacts.

Consistent with findings in other LC target regions,^18–20^ phasic LC activation produced both enhancement and suppression of GC taste responses (Figure 2A). In some cases, LC modulation recruited previously non-responsive neurons (*cell 2529*) or fully suppressed excitatory responses, occasionally producing sign reversals (*cell 1655*) (Figure 2A). Across all significant cell-tastant pairs, individual neurons could be modulated by multiple tastants, but the direction of modulation within each neuron was consistently enhancing or suppressive (Figure 2B). The proportions of LC-enhanced and LC-suppressed neurons were roughly balanced.

### Phasic LC activation enhances GC palatability correlation without altering tastant separability

To determine how LC activation alters GC taste encoding, we analyzed a pseudo-population of LC-modulated, taste-discriminative neurons pooled across mice. To assess population-level taste discriminability, we trained linear decoders to classify tastants using population dynamics. Decoding accuracy increased monotonically with population size; however, accuracy did not differ significantly between stim and no-stim trials (Figure 2C), suggesting that phasic LC activation does not alter overall tastant discriminability in GC.

We next examined palatability encoding using a standard four-tastant panel.^36,51–54^ These tastants differ in hedonic value to mice, with the expected ranking of sucrose > NaCl > citric acid > quinine, which we confirmed using a brief-access taste test (Figure S2). To quantify palatability-related information at the single-neuron level, we calculated the absolute rank correlation (Kendall’s tau) between each neuron’s mean response to the four tastants and their palatability ranking^34,55^ (Figure 2D). Palatability correlation increased gradually following tastant delivery and peaked around 3 s. This time course is delayed relative to prior electrophysiology studies,^33,36^ in which palatability coding peaks ~1–2.5 s after taste delivery, likely reflecting the slower kinetics of calcium imaging. Among LC-modulated neurons, mean palatability correlation was significantly higher in stim than no-stim trials, demonstrating that phasic LC activation enhances palatability coding at the single-neuron level.

### Phasic LC activation reshapes GC population coding along a palatability axis

To examine how LC activation reshapes GC palatability coding at the population level, we performed principal component analysis (PCA) on evoked responses of LC-modulated neurons. Tastant responses formed a low-dimensional manifold organized primarily along a palatability-related axis (Figure 2E). We approximated the palatability axis separately for stim and no-stim conditions as vectors connecting the centroids of palatable (sucrose, NaCl) and aversive (citric acid, quinine) tastants. The most prominent effect of phasic LC activation was a coherent shift in the response manifold that was largely orthogonal to the palatability axes (Figure 2E), which may reflect changes in other GC coding directions (see Discussion).

To isolate LC-induced changes related to palatability coding, we projected responses onto their respective palatability axes (Figure 2F). Phasic LC activation increased the separation between sucrose and quinine, i.e., the dynamic range of the palatability axis, by 1.55-fold. A permutation test confirmed that this increase significantly exceeded that expected from trial-to-trial variability (Figure 2G). We next tested whether the palatability axis itself shifted in orientation and found no change between stim and no-stim conditions (Figure 2G). Both the dynamic range expansion and axis stability persisted when the analysis was extended to the broader population of taste-discriminative neurons (Figures S3A and S3B).

Pairwise tastant distances revealed that this expansion was non-uniform and was driven primarily by increased separation between sucrose and the remaining tastants (Figure S4A). Because palatability axis orientation was stable across conditions, we projected responses onto a common (no stim derived) axis and found that phasic LC modulation shifted NaCl, citric acid, and quinine responses toward an aversive direction, while leaving sucrose unchanged (Figure 2H). Thus, the increased separation between sucrose and other tastants reflects a reorganization of palatability representation rather than a sucrose-specific effect.

To determine whether LC modulation expanded the stimulus manifold beyond the palatability dimension, we quantified dispersion, the mean perpendicular distance from the palatability axis. Phasic LC activation did not significantly alter dispersion, though trended toward expansion (*p* = 0.074; Figure S5A). Together with the non-uniform pairwise tastant distance changes, this indicates that LC modulation does not simply scale the population representation isotropically. Procrustes analysis further showed that LC modulation reorganized tastant centroids into a configuration more closely aligned with palatability order (Figures S5B and S5C).

### Phasic LC activation stretches and rotates GC representations of mixture ratios

Given GC’s role in encoding mixture composition,^56,57^ we examined how phasic LC activation affects responses to sucrose-NaCl mixtures (90:10, 65:35, 35:65, and 10:90). Because sucrose and NaCl differ in palatability (Figure S2), these mixtures likely vary in both mixture-ratio and palatability. Consistent with the palatability experiment, 20.6% of mixture-ratio-discriminative neurons were LC-modulated (Figure S1A), including both LC-enhanced and LC-suppressed subtypes (Figure S6A). Among LC-modulated neurons, mixture-ratio decoding accuracy did not significantly differ between stim and no-stim trials (Figure 3A), indicating that phasic LC activation did not alter overall mixture separability. In contrast, LC activation significantly increased the correlation between neuronal responses and mixture-ratio ranking (Figure 3B), suggesting enhanced encoding of graded mixture differences.

To characterize population-level effects, we performed PCA on mixture responses (Figure 3C). We defined a mixture-ratio axis by connecting the 90:10 and 10:90 centroids and projected responses onto the respective axis. Phasic LC activation increased the dynamic range along the mixture axis (Figures 3C–3E). Pairwise distance analysis showed that this expansion was non-uniform and driven primarily by larger separations between mixtures with greater compositional differences (Figure S4B). LC activation rotated the mixture axis (Figure 3E), possibly reflecting differential stretching of latent dimensions encoding *pure* mixture composition and palatability (see Discussion). Both stretching and axis rotation persisted when analyses were extended to all mixture-ratio-discriminative neurons (Figures S3C and S3D), indicating that the effect was robust across the broader population. Dispersion showed a trend toward reduced separation from the mixture axis (Figure S5D). Together with increased dynamic range, this supports selective elongation of the stimulus manifold along a mixture-relevant axis, as visualized by Procrustes analysis (Figures S5E and S5F).

### Phasic LC activation modestly expands and rotates GC representations of sucrose concentration

We next examined LC’s effect on GC encoding of tastant intensity using four sucrose concentrations (50, 100, 250, and 500 mM). Because sucrose palatability increases monotonically with concentration,^58,59^ these stimuli likely vary in both intensity and palatability. Consistent with prior results, 19.3% of sucrose-concentration-discriminative neurons were LC modulated (Figure S1A), comprising both LC-enhanced and LC-suppressed subtypes (Figure S6B).

Among LC-modulated neurons, phasic LC activation did not significantly alter decoding accuracy or the correlation between neuronal responses and concentration (Figures 3F and 3G), indicating limited modulation of intensity coding. Nevertheless, PCA revealed a stimulation dependent increase in dynamic range along the sucrose-concentration axis (Figures 3H–3J). Pairwise distance analysis showed only modest changes, with only the 250 versus 50 mM comparison surviving correction (Figure S4C), consistent with relatively weak dynamic-range expansion. LC modulation also induced a significant rotation of the concentration axis (Figure 3J), possibly reflecting differential stretching of latent dimensions encoding concentration and palatability. When analyses were extended to all sucrose-concentration-discriminative neurons, axis rotation persisted, whereas dynamic-range stretching was no longer significant (Figures S3E and S3F), suggesting that axis rotation is the more robust population-level effect. Dispersion showed a significant LC-induced expansion orthogonal to the concentration axis (Figures S5G–S5I), possibly reflecting non-linear concentration coding not captured by the approximated axis.

### Parsing tuning modulations underlying population-level effects of phasic LC activation

The preceding analyses showed that phasic LC activation induces distinct geometric transformations of GC population activity, including (but not limited to) dynamic-range expansion and axis rotation (Figure 4A). Because representational geometry is determined by neuronal tuning,^31^ we next examined LC-induced tuning changes underlying these transformations. We defined each neuron’s tuning profile as its mean response across a tastant set, and, using a model comparison approach, classified LC-induced changes as scale-shift modulation, flexible modulation, or no modulation for each experiment (Figure 4B). Scale-shift modulation reflects a uniform transformation of the tuning profile, captured by a common multiplicative factor and additive offset across tastants. In contrast, flexible modulation reflects tastant-specific changes that cannot be explained by uniform scaling or shifting. Example scale-shift and flexible neurons from the palatability experiment are shown in Figures 4C and 4D, respectively. Scale-shift modulation preserved the relative response pattern across tastants, whereas flexible modulation could reorganize it, including changes in rank order. Across experiments, basic-taste-discriminative neurons showed more scale-shift modulation and less flexible modulation than mixture-ratio- and sucrose-concentration-discriminative neurons (Figures 4E, 4H, 4K, and S7A).

### LC-mediated gain modulation is structured and attribute specific

We next examined the additive and multiplicative factors associated with scale-shift-modulated neurons, given their potential contribution to population-level transformations. Additive factors shift the population manifold without changing its shape or size, whereas multiplicative factors can expand, compress, and/or rotate it. Both additive and log-transformed multiplicative factors spanned positive and negative values (Figure S7B). Median log-multiplicative factors were positive across all experiments, indicating an overall bias toward expansion (Figure S7C). Multiplicative effects were less expansive in the sucrose-concentration experiment than in the mixture experiment, consistent with the more modest expansion of sucrose dynamic range.

Given the relevance of multiplicative effects (gain modulation) to manifold expansion, we next examined whether multiplicative factors related to each neuron’s contribution to the coding axis, measured by its absolute axis weight. No correlation was observed in the palatability experiment (Figure 4F), whereas significant negative correlations emerged in the mixture-ratio and sucrose-concentration experiments (Figures 4I and 4L). Thus, under phasic LC modulation, neurons contributing less strongly to these coding axes were preferentially expanded, whereas stronger contributors were relatively compressed.

### Distinct contributions of scale-shift and flexible modulation to LC-induced transformations

We next applied PCA separately to scale-shift and flexibly modulated neurons to determine their contributions to coding-space transformations. Scale-shift modulation increased dynamic range in the palatability and mixture-ratio experiments (Figures 4G and 4J), but not in the sucrose-concentration experiment (Figure 4M), consistent with the stronger expansion bias of multiplicative factors in the former two datasets (Figure S7C). Despite its potential to reweight neuronal contributions, scale-shift modulation did not induce axis rotation in any experiment. Thus, scale-shift modulation primarily contributed to LC-induced dynamic-range expansion.

In contrast, flexible modulation induced significant axis rotation across all experiments (Figures 4G, 4J, and 4M), consistent with its ability to reshape neuronal tuning. Flexible modulation also increased dynamic range in the sucrose-concentration experiment, but not in the palatability or mixture-ratio experiments. Analysis of rank correlations revealed that scale-shift modulation increased response-stimulus correlation in the palatability and mixture experiments (Figures S7D, S7F, and S7H), whereas flexible modulation increased these correlations across all attributes (Figures S7E, S7G, and S7I). Together, these findings indicate that scale-shift and flexible modulation make distinct contributions to LC-induced population transformations, with the former primarily supporting dynamic-range expansion and the latter driving axis rotation.

### Tonic LC activation has limited impact on GC taste encoding

Previous work suggests that phasic and tonic LC activation can produce qualitatively different effects on downstream circuits.^26,28,60^ To examine the impact of tonic LC activation on GC taste encoding, we conducted three additional experiments using tastants varying in palatability, mixture ratio, and sucrose concentration, paired with an alternative stimulation protocol. Tastant trials were divided into eight blocks, half of which included 5-Hz tonic LC stimulation (Figure 5A). Validation experiments confirmed that this protocol elicited robust NE release that persisted throughout each stimulation block (Figures 5B and 5C).

Despite robust NE release, tonic LC activation modulated only ~10%–12% of stimulus-discriminative neurons across attributes (Figure S1B), approximately half the proportion observed under phasic stimulation (Figure S1A), though still higher than in tdTomato controls. Tonic activation did not affect palatability or mixture-ratio decoding but reduced decodability for sucrose concentration (Figure S8). Tonic activation also did not alter response-stimulus correlations for any taste attribute (Figure S8). PCA showed that tonic LC activation produced axis rotation across all attributes without changing dynamic range (Figures 5D–5I). When all stimulus discriminative neurons were included, rotation persisted for mixture ratio and sucrose concentration, but not palatability (Figure S9). Together, these findings suggest that, unlike phasic activation, tonic LC activation produced modest transformations that are not clearly aligned with behaviorally relevant stimulus dimensions.

## DISCUSSION

Here, we examined how LC activation shapes cortical taste encoding at single-neuron and population levels. A central advance of this study is that it links LC activation patterns to specific geometric transformations of gustatory cortical population activity, revealing how a major neuromodulatory system can reorganize taste representations across multiple stimulus dimensions. Phasic LC activation (1) bidirectionally modulated taste-evoked responses, with each neuron showing a consistent direction of modulation, and (2) enhanced correlations between neuronal responses and graded stimulus properties in the palatability and mixture-ratio experiments. At the population level, phasic LC activation (1) stretched the dynamic range of GC activity along attribute-relevant axes and (2) rotated the mixture-ratio and sucrose-concentration axes, without affecting the palatability axis. Dynamic-range stretching was driven by structured multiplicative modulation, whereas axis rotation reflected flexible reshaping of neuronal tuning. In contrast to phasic LC activation, tonic activation influenced fewer GC neurons and produced axis rotation without dynamic-range stretching. Together, these results indicate that brief LC activation reorganizes GC population coding in a pattern-dependent manner, providing understanding of how neuromodulation biases behaviorally relevant taste representations, particularly along the palatability dimension.

### Population-level effects of LC modulation

The geometry of population activity determines how sensory information is represented. Expansion along an attribute-relevant axis is linked to increased perceptual separation along that dimension, and selective attention can stretch neural representations along task-relevant axes.^61,62^ Although LC activity is more commonly associated with global arousal than with top-down control, it may nonetheless bias information processing toward specific sensory attributes. Consistent with this idea, phasic LC activation primarily increased the separation between sucrose and other basic tastes along the palatability dimension without expanding orthogonal dimensions, suggesting selective reorganization of palatability coding. The absence of expansion along other taste-differentiating dimensions may help explain the unchanged decoding performance under LC modulation. These findings are consistent with prior work showing that behavioral state differentially influences taste identity and palatability representations in GC.^37,38^ Notably, the palatability shift was driven mainly by aversive shifts in NaCl, citric acid, and quinine responses, whereas sucrose responses remained largely unchanged. This effect differs from other forms of palatability reorganization previously observed in GC. For instance, task disengagement increases the separation between appetitive and aversive tastants,^37^ while familiarization clusters appetitive tastants.^63^ In our study, the aversive shift in all tastants except the most palatable one could bias animals toward selecting highly palatable foods, a potentially adaptive strategy when navigating unfamiliar foraging environments. The relative stability of sucrose representations may relate to its caloric value, a possibility that could be tested by comparing LC modulation of sucrose and non-caloric sweeteners.

Axis rotation provides an additional way to alter population geometry. The rotation of the mixture and sucrose-concentration axes admits several interpretations that do not require alterations in downstream readout. One interpretation is that these axes reflect blending of two latent dimensions: objective stimulus properties and palatability. If LC modulation scales these dimensions unequally, the resulting attribute axis would rotate toward the dimension undergoing greater expansion. An intriguing possibility is that the latent palatability dimension underwent preferential expansion, which could explain the population-level transformation observed across all three phasic stimulation experiments. This interpretation is also consistent with our observation that multiplicative modulation expands low-contribution neurons while compressing high-contribution neurons. If these groups preferentially represent distinct latent dimensions, such opposing gain modulation would reweight those dimensions. Alternatively, LC activation may recruit previously non-discriminative neurons into stimulus encoding, effectively pulling the attribute axis toward the subspace they span. These neurons would likely be classified as flexibly modulated, consistent with their role in both driving axis rotation and increasing response-stimulus correlations. Importantly, axis estimates could be sensitive to noise, particularly when the defining centroids are close together, and thus the precise angle should not be interpreted literally. This variability is addressed here by assessing significance through permutation testing.

Beyond stretching and rotation, phasic LC activation produced a sizable coherent shift of the response manifold largely orthogonal to the palatability axis, which is likely driven by the additive (shifting) components of scale-shift modulation. Although this shift could reflect another taste-related coding dimension, we did not observe large displacements aligned with the mixture-ratio or sucrose-concentration axes. Alternatively, the LC-induced shift orthogonal to the palatability axis may reflect an internal-state dimension, such as arousal, that is linked to LC output.^64,65^ This shift may also represent causal or compensatory changes in the network that enable or accompany expansion and rotation in taste-relevant coding dimensions.

### Mechanisms of LC-induced neuronal effects

LC modulation can shape cortical taste responses through adrenergic receptor-mediated effects on GC neurons, local recurrent GC circuits, and/or other brain regions that influence GC. Both glutamatergic and GABAergic GC neurons express transcripts for several adrenergic receptors, including the α1-, α2-, and β1-receptor subtypes.^66^ Activation of these receptors can influence intrinsic and synaptic properties of cortical neurons,^67–71^ potentially contributing to the enhancement, suppression, and diverse tuning effects observed here. LC output may also influence neuronal responses indirectly through its effects on other cortical microcircuit components, including inhibitory neurons, which were not examined in this study. Additionally, changes in GC activity may reflect LC’s impact on other nodes of the taste pathway, such as the gustatory thalamus and basolateral amygdala, which primarily convey chemosensory^72–74^ and hedonic information,^52,75,76^ respectively. Thus, our results may reflect both local adrenergic modulation within GC and LC-driven changes across the broader gustatory network. How LC influences GC neurophysiology and inter-regional interactions remains an important open question.

Characterizing whether LC enhances or suppresses GC responses to different tastants alone is not sufficient to explain population reorganization. Population-level effects such as palatability-axis expansion and coding-axis rotation depend on how tuning profiles are reshaped. To examine LC-mediated tuning changes, we examined scale-shift and more flexible modulatory effects. Decomposing modulation into additive and multiplicative components (scale-shift) is a common framework for characterizing modulatory effects on neuronal tuning.^77–79^ Multiplicative (gain) modulation, in particular, is a well-established mechanism by which the LC-NE system can facilitate cognition and information processing.^11,80–82^ Although the cellular mechanisms underlying additive and multiplicative modulation have been extensively studied,^79,83,84^ the basis of flexible modulation remains less clear, given its heterogeneous nature. Recruitment of coding neurons, a form of flexible modulation described above, may reflect unmasking of attribute-relevant inputs. Alternatively, flexible modulation may arise from differential scaling of narrowly tuned, labeled-line-like inputs, effectively altering the relative contribution of distinct sensory streams. Importantly, our modulation classes are descriptive rather than mechanistic. This framework provides a way to relate diverse single-neuron tuning changes to the reorganization of GC population coding induced by LC activation.

### Links to state-dependent modulation

Prior work has shown enhanced GC palatability coding during task disengagement^37^ and LiCl-induced sickness.^38^ Both behavioral conditions are accompanied by shifts in cortical state reflected in local field potential patterns. Because LC-NE activity is tightly coupled to cortical state^85,86^ and can rapidly modulate it,^87–90^ LC signaling may provide a route through which internal state influences sensory coding by altering cortical state. Whereas these prior studies examined behavioral states lasting tens of minutes, our results show that gustatory representations can be reorganized on the timescale of transient LC activation, contributing to moment-to-moment variability in sensory responses.^4^

Understanding LC function requires moving beyond general labels such as “arousal” to consider specific LC dynamics in sensory-guided behaviors. In the context of taste, prior work shows that LC phasic activation occurs immediately before consumption^42^ and during brief gaps between lick bouts^45^; these dynamics were mimicked by our phasic stimulation pattern and may transiently prime GC before each influx of gustatory input. LC activity is also suppressed *during* consumption bouts,^12,42,45^ potentially stabilizing sensory representations by reducing LC-driven fluctuations. More broadly, many learning paradigms deliver gustatory rewards immediately after reward-seeking actions, so phasic LC activation accompanying these actions^13,14,91^ may extend into reward processing and influence learning.

The distinct effects of phasic and tonic LC activation highlight the importance of activation pattern. A classic framework proposes that moderately aroused states, associated with phasic LC activation, optimize information processing, whereas hyper-aroused states, associated with high tonic LC activation, do not.^2,11,92^ Although prior studies have shown that phasic and tonic LC activation can differentially affect sensory processing,^26,28^ our results show that the sensory-enhancing effects of phasic LC activation on GC neurons were not evident under high tonic activation. However, these differences may also reflect stimulation timing, because tonic stimulation occurred throughout the experiment whereas phasic stimulation occurred selectively before tastant delivery. The timing of LC activity relative to sensory stimuli has been proposed to partially account for the differential effects of phasic and tonic LC stimulation on sensory processing.^93^ Future experiments comparing the same LC stimulation pattern before versus during tastant delivery will be needed to isolate timing effects relative to sensory input. Tonic LC activation reduced decodability only for sucrose-concentration-coding neurons, suggesting a selective diminishment in intensity representation. A proposed mechanism for divergent effects of LC activity patterns is that phasic activation preferentially engages high-affinity α1 receptors through moderate NE release, whereas sustained tonic activity recruits lower-affinity β receptors with distinct cellular consequences.^94^ Consistent with our findings, recent fMRI work suggests that phasic LC activation produces brain-wide interaction patterns that may favor sensory processing relative to tonic activation.^60^Together, these findings suggest that LC’s influence on sensory coding depends on its activation pattern.

### Open questions

Our work also provides a foundation for probing more advanced aspects of population coding, including response dynamics and noise correlations. GC taste responses unfold as sequences of ensemble states that encode presence, identity, and palatability^33,34,95^; how LC activity shapes these transitions remains unknown and will likely require higher-temporal-resolution-recordings, such as electrophysiology. Another open question is how LC affects noise correlations—the shared trial-to-trial variability between neurons—which influence population information capacity^96–98^ and are regulated by internal state^99^ and neuromodulation.^100^ Addressing this will require simultaneously recorded populations and larger numbers of trials than used here. In addition to encoding basic properties of taste stimuli, GC dynamics contribute to taste expectation,^54,101^ multisensory integration,^102–105^ and taste-guided decision making.^57,106,107^ By revealing how LC reshapes GC taste representations, our findings provide a basis for future studies to understand how state-dependent neuromodulation influences the neural computations underlying natural feeding behavior.

### Limitations of the study

Several limitations should be considered when interpreting our findings. First, whether LC-induced shifts in GC population responses reflect arousal remains to be tested. Future experiments could examine taste-evoked responses during natural arousal fluctuations monitored with EEG or pupil measures to test whether this orthogonal shift is reproduced. Second, the behavioral consequences of LC-induced coding reorganization remain untested. A full assessment will require brief-access,^58^ two-bottle choice,^108^ and taste-reactivity^109^ assays to measure consummatory behavior, taste preference, and affective orofacial reactions across sex-balanced cohorts, as these behaviors are known to exhibit sex differences.^110–112^ Third, our interpretation of axis rotation as reflecting unequal stretching of covarying taste attributes remains speculative. A stronger test would present the mixture-ratio or concentration stimulus sets together with the standard four-taste panel in the same session, allowing the latter to provide a stable palatability reference axis. Fourth, the effects observed in each experiment may depend on the tastant battery, including stimulus selection and concentration range. Finally, because our population-level analyses rely on distinct attribute axes defined in separate PCA spaces for each stimulus set, direct statistical comparisons *across* experiments are not available. Therefore, differences among the palatability, mixture-ratio, and sucrose-concentration results should be interpreted cautiously.

## STAR★METHODS

Detailed methods are provided in the online version of this paper and include the following:

### EXPERIMENTAL MODEL AND STUDY PARTICIPANT DETAILS

Adult male and female mice older than postnatal day 60 (>P60) were used in all experiments. *Dbh*^*Flpo*^ mice^17^ were maintained as heterozygotes on a C57BL/6J background. Mice were housed under a reversed 12-h light/dark cycle, with lights off at 9 A.M. (zeitgeber time 0, ZT0), and all experiments were conducted during the dark period. Mice had *ad libitum* access to food and water except during experiments requiring water restriction. Prior to surgery, mice were group housed. Both male and female mice were included, but the study was not designed or powered to assess sex-dependent effects, which therefore remain a limitation. All animal procedures were approved by the University of Connecticut Institutional Animal Care and Use Committee (IACUC) and were conducted in accordance with the *Guide for the Care and Use of Laboratory Animals*.

### METHOD DETAILS

#### Survival surgical procedures

All survival surgical procedures were performed under 1–4% isoflurane anesthesia using aseptic techniques. Mice were positioned in a stereotaxic frame (Model 942, Kopf Instruments) atop a warming pad (RT-0514, Kent Scientific) to maintain body temperature. Anesthesia was delivered via a nose cone and continuously monitored throughout the procedure to ensure proper depth. For analgesia, ketoprofen (5 mg/kg i.p., Covetrus) was administered during surgery and again at 24- and 48-h post-operatively for all procedures.

#### Intracranial viral injections

Viral constructs were packaged in the indicated serotypes, and titers are reported as genome copies per milliliter (GC/mL) following dilution in physiological saline. Intracranial injections were performed after a midline scalp incision, followed by skull exposure and leveling. Unilateral craniotomies were made using a surgical drill (OmniDrill 35, WPI). Viral solutions were loaded into glass micropipettes pulled from capillary tubes (Fisher Scientific, Part 21171), backfilled with mineral oil (Fisher Scientific, Part O1211), and connected to a Nanoliter 2020 injector (WPI).

For miniscope imaging with LC activation, *Dbh*^Flpo^ mice received unilateral LC injections of AAVs encoding either the Flp-dependent optogenetic activator ChrimsonR^42,113^ (AAV-Ef1a-fDIO-ChrimsonR-tdTomato; serotype 5; 5 × 10^12^ GC/mL; Lot #: 09012021; *n* = 13 mice) or a fluorescent control^42^ (AAV-Ef1a-fDIO-tdTomato; serotype 5; 2.9 × 10^12^ GC/mL; Lot #: 09012021; *n* = 3 mice). In the same surgery, a CaMKIIa-driven calcium indicator (GCaMP8m) was injected in the ipsilateral GC using AAV-CaMKIIa-jGCaMP8m-WPRE (serotype 9; 1.15 × 10^13^ GC/mL; Lot #: V197511) to target pyramidal neurons. For LC injections, 500 nL was delivered at 100 nL/min. Coordinates, relative to bregma were: anterior-posterior (AP) −5.45 mm, medial-lateral (ML) −1.25 mm, and dorsal-ventral (DV) −3.85. We used unilateral LC manipulation because LC projections to cortex are predominantly ipsilateral (~95%).^114^ For GC injection, 500 nL was delivered at 50 nL/min. Coordinates, relative to bregma: AP +1.2 mm, ML −3.3 mm, DV −1.95 (from brain surface).

To monitor NE dynamics in the GC during LC activation, a separate cohort of *Dbh*^Flpo^ mice (*n* = 3 mice) received unilateral AAV injections of Flp-dependent ChrimsonR^42,113^ into the LC and a 4:1 cocktail of AAVs expressing the constitutive NE biosensor GRAB_NE2h_^43^ (AAV-hSyn-GRAB-NE2h; serotype 9; titer: 8 × 10^12^ GC/mL; Lot #: 20240529) and tdTomato (AAV-CAG-tdTomato; serotype 9; titer: 4.0 × 10^10^ GC/mL; Lot #: V197427) into the ipsilateral GC. For this GC injection, 500 nL of the viral cocktail was infused at 100 nL/min. Coordinates relative to bregma: AP +1.2 mm, ML −3.3 mm, DV −2.0 (from brain surface).

After each injection, the micropipette was left in place for 5 min to minimize backflow. Incisions were sutured, and mice recovered on a heating pad for at least 24 h.

#### Intracranial optical fiber and grin lens implantation

All procedures preceding viral injection, including pre-operative care, were conducted as previously described. This section outlines the implantation of custom fiber-optic probes and gradient refractive index (GRIN) lenses. Fiber-optic probes were constructed using 200 μm, 0.39 NA multimode fiber (FT200EMT, Thorlabs) housed in ceramic ferrules (MM-CON2007–2300, Precision Fiber Products) and were used for optogenetic stimulation in the LC or NE fluorescence detection in the GC. GRIN lenses (ProView Integrated Lens, 1 mm Ø × 4 mm length, with baseplate; Inscopix) were used for GC imaging.

For mice expressing either ChrimsonR or tdTomato in the LC, along with GCaMP8m in GC, a second surgery was performed ≥ three weeks after viral injection to implant the optical components. A small craniotomy was made above the LC and a larger one above the GC using a 1.4 mm inverted-cone bur (Panther). An optical fiber was unilaterally implanted above the LC at coordinates AP: −5.45 mm, ML: −1.25 mm, DV: −3.85 mm relative to bregma, and secured to the skull using Metabond (S371, S398, and S396, Parkell), while leaving the GC craniotomy open.

To guide GRIN lens placement above the GC, a flattened needle was lowered to 1.65 mm below the brain surface and then withdrawn. The GRIN lens was then implanted at 1.80 mm depth and secured with Metabond. For mice expressing ChrimsonR in LC and GRAB_NE2h_ in GC, a second surgery was performed three weeks post-injection to implant optical fibers above both regions. The LC fiber was implanted at AP −5.45 mm, ML −1.25 mm, DV −3.85 mm (bregma-referenced), and GC at AP +1.2 mm, ML −3.3 mm, DV: −2.0 mm (relative to the brain surface). Incisions were sutured, and post-operative care followed the protocol described above.

#### Intraoral cannulae (IOC) implantation

A third surgery was performed to implant IOC in mice for miniscope recordings, at least two weeks after optical implantations. Each IOC consisted of a 1.5 cm segment of plastic tubing (1883T3, McMaster-Carr), with one end flared to secure a trimmed plastic washer (93785A812, McMaster-Carr). Mice were anesthetized via subcutaneous (S.C.) injection of ketamine (100 mg/kg) and xylazine (12.5 mg/kg) mixture (Covetrus).

A scalp incision was made to expose the lateral edge of the skull. A needle was attached to the non-flared end of the IOC, which was inserted lateral to the second/third maxillary molar, passed through the masseter muscle and beneath the zygomatic arch, and exited at the scalp edge behind the eye. After removing the needle, the exposed end of the IOC was connected to a push-in fitting (QSM-M3–3, Festo). Mice were placed in a stereotaxic frame equipped with a heating pad and maintained on 1–4% isoflurane anesthesia. The IOC was secured to the skull using Metabond and dental acrylic, and the incision was sutured closed. A fishing line plug was inserted into the IOC to prevent obstruction. To prevent infection, Penicillin G procaine (3,000 units/lb., Norbrook; 0.01 to 0.02 mL; intramuscular) was administered immediately post-surgery and again 48 h later. All other post-operative care followed the protocol described above.

#### Water restriction protocol

Following IOC surgery, mice were initially provided with water *ad libitum*. Starting at least two weeks post-surgery, they were gradually transitioned to a 23-h water restriction protocol to stabilize fluid intake prior to neural recordings. Acclimation occurred over three days, with restriction periods progressively increasing from 12 to 23 h. Once acclimated, mice received a minimum of one hour of water access daily, after passive tastant delivery (described below), to account for potential post-experiment satiety. Body weight and general health were monitored and recorded throughout restriction, which was limited to a maximum of two weeks.

#### Passive tastant delivery

Taste stimuli were delivered using a gravity-driven, valve-controlled perfusion system (VC3–4PG, ALA Scientific Instrument). This system included four fluid reservoirs, each connected via separate 1/16″ PVC tubing (MMF-4, ALA Scientific Instruments) that converged at a custom-made manifold composed of four jointed miniature polyimide tubes (085-III, MicroLumen). Before each experiment, the IOC plug was removed, and the cannulae was cleaned using a fishing line to dislodge debris, followed by rinsing with deionized water-filled syringe, and flushing with compressed air to prevent obstruction.

To ensure consistent tastant delivery, mice were briefly anesthetized with 4% isoflurane, and any food debris or scar tissue near the IOC opening was removed with forceps. Mice were then placed in an empty arena and allowed to recover for at least 20 min. Immediately before each experimental session, the manifold was inserted into the IOC and secured using the push-in fitting. The manifold was designed so that its tip aligned flush with the IOC opening.

Three sets of taste stimuli were tested in separate experiments, including: 1) *Basic tastes*: 200 mM sucrose, 50 mM NaCl, 20 mM citric acid, and 1 mM quinine, 2) *Sucrose concentrations*: 50 mM, 100 mM, 250 mM, and 500 mM, and 3) *Sucrose-NaCl mixtures*: 100 mM sucrose and 100 mM NaCl mixed at ratios of 90:10, 65:35, 35:65, and 10:90. Each experiment consisted of 96 trials with tastants presented in pseudo-random order with intertrial intervals of 25–35 s. During each trial, a solenoid valve opened for 0.08 s to deliver 8 μL of tastant. The perfusion system was rinsed and calibrated daily to ensure consistent fluid delivery. We did not include water rinses between tastant deliveries, consistent with prior studies^36,38,52,76^ because awake mice rapidly clear tastants through salivation. Any residual cross-trial tastant interactions would be expected to increase trial-to-trial variability rather than introduce systematic bias across conditions.

#### *In vivo* miniscope calcium imaging

To monitor large-scale, single-cell Ca^2+^ activity in the GC of freely moving mice, we used the nVoke 2.0 miniature microscope system (Inscopix). The miniscope was secured to the baseplate by tightening a small set screw and connected to a commutator (Inscopix), which enabled high-speed video and signal transmission while minimizing cable entanglement. The commutator was linked to a data acquisition (DAQ) box (Inscopix).

On the day of the experiment, mice were placed in an operant chamber (ENV-370W, Med Associates) within a sound-attenuating cubicle (ENV-017M, Med Associates) and given 15 min to habituate. The miniscope data were collected through the Inscopix Data Acquisition Software (IDAS). The miniscope has an excitation blue LED (455 ± 8 nm) that was set to 0.2 arbitrary unit power. The imaging gain (ranging from 2.0 to 3.0) was adjusted based on the fluorescence intensity histogram to ensure that the highest possible dynamic range was achieved without signal saturation. Data were collected at 20 frames per second (fps) with ~50 ms exposure time. Synchronization of calcium imaging with tastant delivery and optogenetic stimulation was achieved using MedPC scripts (Med Associates) and transistor–transistor logic (TTL) signals delivered at 20 Hz.

#### Optogenetic stimulation procedures

To activate LC neurons expressing ChrimsonR, a 532 nm green laser (Shanghai Laser & Optics Century Co., Ltd., China) was coupled to an optical patch cable (M72L02, M83L01, Thorlabs) and connected to the implantable probe via a mating sleeve (ADAL4–5, Thorlabs), which was wrapped in tinfoil to prevent light leakage. Laser power was adjusted to 8 mW at the fiber tip.

Two photostimulation paradigms were employed. In the *phasic stimulation* paradigm, half of the tastant delivery events were preceded by a 0.5-s train of laser pulses (20 Hz, 5 ms pulse duration). Tastants were administered 0.5 s after the photostimulation train ended. In the *tonic stimulation* paradigm, a total of 96 tastant delivery events were organized into eight blocks. Photostimulation at 5 Hz was applied during four of these blocks in the following sequence: on, off, off, on, on, off, off, on. Within each stimulation block, the tonic pulse train began 45–55 s before the first tastant delivery and continued until 10 s after the final delivery. The subsequent block began 145–155 s after the preceding stimulation ended.

#### Experimental timeline

Two weeks following IOC implantation, mice were subjected to 23-h water restriction to enhance motivation for tastant sampling. Prior to recording sessions, mice were habituated to wearing the miniscope during passive taste delivery in the testing chamber for three days. Each habituation session consisted of 96 tastant trials, including 200 mM sucrose, 50 mM NaCl, 20 mM citric acid, and 1 mM quinine, allowing mice to become familiar with the stimuli.

The experimental design combined three sets of taste stimuli with two optogenetic stimulation paradigms, resulting in six distinct conditions: 1) Basic tastes–phasic stimulation, 2) Sucrose concentrations–phasic stimulation, 3) Mixtures–phasic stimulation, 4) Basic tastes–tonic stimulation, 5) Sucrose concentrations–tonic stimulation, and 6) Mixtures–tonic stimulation. These conditions were tested in the order listed above. Experimental sessions were occasionally separated by rest days, during which mice received 1 h of water access without undergoing testing.

#### Fiber photometry recordings of photostimulation-evoked NE release

Five weeks post-optical fiber implantations, *in vivo* fiber photometry recordings were conducted to measure NE release in the GC following optogenetic stimulation of LC neurons. Recordings were conducted using a custom-built spectrally resolved fiber photometry system.^42,44,45^ Excitation light at 488 nm (OBIS 488LS-20, Coherent) was adjusted to ~60 μW power and directed into a fluorescence cube (DFM1, Thorlabs), reflected by a dichroic mirror (ZT488/561rpc-UF1, Chroma), and focused via an achromatic fiber port (PAFA-X-4-A, Thorlabs) onto a patch cable (M83L01, Thorlabs). The distal end was connected via a quick-release interconnect (ADAL3, Thorlabs) to an optical probe mounted on the mouse skull.

Fluorescence emitted from the tissue traveled back through the same optical path, passed through the dichroic mirror, and was filtered (ZET 488/561m, Chroma). The filtered signal was routed through a fiber port (PAF2S-11A, Thorlabs) and transmitted to a spectrometer (Ocean FX, Ocean Optics) via patch cable (M200L02-A, Thorlabs). Time-resolved fluorescence emission spectra of GRAB_NE2h_ and TdTomato signals were acquired using OceanView software (version 2.0.8) at 25 frames per second, with a 31 ms integration time. Synchronization of calcium imaging and optogenetic stimulation was achieved using MedPC (Med Associates) scripts and TTL signals delivered at 25 Hz.

Two recording sessions were conducted to test distinct photostimulation paradigms for activating ChrimsonR-expressing LC neurons and their effects on NE release in GC. In the first session, mice received *phasic stimulation,* consisting of 0.5-s trains of light pulses (5-ms pulse duration; 532 nm) delivered at 20 Hz. In the second session, mice received *tonic stimulation* at 5 Hz pulses. Both stimulation conditions used the same trial structure as the phasic and tonic miniscope experiments, respectively, except without tastant delivery.

#### Brief-access taste testing

Palatability of the four tastants used in the palatability experiment was assessed using a brief-access taste test. Eight *Dbh*^Flpo^ mice (separate from the above experimental subjects) were water restricted for 23 h prior to testing. During testing, mice licked a metal spout fitted with four microtubes, each connected to a reservoir containing one tastant solution. Licks were detected with a lickometer (Med Associates ENV-250), and each lick triggered delivery of ~1 μL of tastant via the opening of a solenoid valve.

A trial began with the mouse’s first lick on the spout; the spout remained accessible for 10 s before being retracted. After a 10-s inter-trial interval, the spout was extended again to allow initiation of the next trial. Each session lasted 30 min, during which tastants were delivered in pseudo-random order and lick timestamps were recorded. The tastants tested—200 mM sucrose, 50 mM NaCl, 20 mM citric acid, and 1 mM quinine—were identical to those used in the palatability experiment.

#### Tissue collection and processing

At the conclusion of the experiments, brain tissue was collected from all mice to verify viral expression and optical implant placement. Mice were deeply anesthetized with isoflurane and perfused transcardially with 1% phosphate-buffered saline (PBS) containing heparin (1:1000), followed by 4% paraformaldehyde in PBS (PFA/PBS), also supplemented with heparin. Brains were post-fixed overnight in 4% PFA/PBS at 4°C on a shaker, then cryoprotected in 30% sucrose in PBS for 48 h. Following cryoprotection, brains were embedded in optimal cutting temperature (O.C.T.) compound (Thermo Fisher), frozen, and stored at −80°C. Coronal sections (40-μm) were cut using a cryostat (Leica CM 3050S) and transferred into a cryoprotectant storage solution containing 30% sucrose, 30% ethylene glycol, and 40% 0.1 M phosphate buffer. Sections were stored at −80°C until further processing.

#### Immunohistochemistry

Brain sections were rinsed in 0.1 M PBS and blocked for 1 h at room temperature on a shaker in 5% normal goat serum made in 0.1% Triton PBS. Sections were then incubated overnight at 4°C in primary antibodies, including chicken anti-GFP (1:10,000; Abcam; Lot # GR3361041–14), rabbit anti-DsRed (1:1000; Takara; Lot # 2103116), and mouse anti-TH (1:500; GeneTex; Lot # 822104279). The next day, tissues were washed in 0.1% Triton X-100 dissolved in 0.1 M PBS and incubated for 2 h at room temperature on a shaker with secondary antibodies (1:1000; Fisher), including goat anti-chicken Alexa 488 (Lot # 2304258), goat anti-rabbit Alexa 568 (Lot # 2273773), and goat anti-mouse Alexa 633 (Lot # 2304276). After final rinses in PBS, brain sections were mounted onto slides and cover slipped using Prolong Diamond Anti-Fade mounting medium with DAPI (4^′^,6-diamidino-2-phenylindole; P36971, Thermo Fisher).

#### Digital image processing

Immunofluorescent-labeled brain sections were imaged at 10× magnification using a Leica DM6 epifluorescent microscope (Leica Microsystems, Wetzlar, Germany). To enhance image clarity and reduce out-of-focus blur, all DM6 images were deconvolved using the Leica Instant Computational Clearing algorithm (LAS X 3.8.1). High-magnification images of the regions of interest were acquired at 40*x* using a Lecia SP8 spectral confocal microscope (Leica Microsystems). Digital images were exported from LASX software (v3.7.5) as uncompressed .lif or .tif files and opened in FIJI (v2.0) for further analysis. z-stacks were converted to maximum-intensity projections, and image adjustments were limited to uniform brightness and contrast changes to enhance fluorescent signal visibility.

#### Data presentation and visualization

All graphical illustrations were made using Adobe Illustrator, Photoshop, GraphPad Prism, and MATLAB.

### QUANTIFICATION AND STATISTICAL ANALYSIS

Data analysis was performed using custom scripts written in MATLAB (MathWorks) and Prism 10 (GraphPad). Unless otherwise indicated, statistical significance in figures is denoted as *p < 0.05, **p < 0.01, and ***p < 0.001; n.s. indicates *p* ≥ 0.05.

#### Miniscope movie pre-processing and cell-identification

Miniscope recordings were pre-processed using Inscopix Data Processing Software (IDPS), including spatial and temporal down-sampling, bandpass filtering, and motion correction. Cell bodies were identified using constrained non-negative matrix factorization (CNMFe),^115^ producing fluorescence traces in units of dF/noise. Extracted cells and traces were visually inspected to remove poorly identified cells. The traces were down-sampled to 10 Hz.

#### Identification of taste-discriminative and LC-modulated neurons

All analyses were performed in MATLAB unless noted otherwise. Neuronal activity was aligned to tastant delivery and baseline-corrected by subtracting the mean response during the 1–4 s window preceding delivery. Aligned responses were pooled across subjects to construct pseudo-populations. Analyses focused on the 5 s post-tastant delivery period, divided into ten 500-ms time bins.

To identify stimulus-discriminative neurons, repeated-measures (RM) two-way ANOVAs (tastant × time bin) were performed on each neuron. Neurons were classified as stimulus-discriminative if either the tastant main effect or the tastant × time interaction was significant (*p* <0.05, two-tailed). To ensure robust evoked responses, we applied two additional criteria: (i) paired *t*-tests comparing each post-delivery time bin with baseline, retaining neurons with at least one significant time bin at *p* <0.001; and (ii) mean evoked response that differed from baseline by ≥1 dF. This procedure was applied separately to stim and no-stim trials, and neurons identified in either condition were combined. Thus, neurons were included in the stimulus-discriminative category if they met criteria in either condition, including cases near the classification boundary.

LC-modulated neurons were identified using RM two-way ANOVAs (stim-condition × time bin) separately for each tastant. Neurons were classified as LC-modulated if either the stim-condition main effect or interaction reached significance (*p* <0.01) for at least one tastant. The proportion of LC-modulated neurons in experimental and tdTomato control mice was compared using exact binomial tests (performed in R).

#### Population decoding

To decode tastant identity, linear discriminant classifiers (MATLAB *fitcdiscr* function) were trained on time-bin-resolved population responses. Decoding accuracy was evaluated via 6-fold cross-validation. To estimate performance for a given population size, 100 subpopulations were constructed by sampling neurons with replacement, and mean accuracy was computed. To test differences between stim and no-stim conditions, trial labels were permuted 1,000 times for each population size to generate a null distribution. The observed decoding difference was compared to the null distribution (two-tailed *p*-value). Significance across population sizes required at least three sizes with *p* <0.05. If this criterion was not met, no significance (n.s.) was noted on graphs.

#### Response–stimulus correlation

Attribute-relevant information at the single-neuron level was quantified by computing Kendall’s rank correlation coefficient (Kendall’s tau) between each neuron’s mean responses to tastants and the ranked attribute values. LC stimulation effects were assessed by comparing the absolute values of tau across stim and no-stim conditions using RM two-way ANOVA (stimulation × time) followed by Bonferroni *post hoc* tests (using GraphPad Prism).

#### Principal component analysis (PCA) and LC-induced transformations

Because PCA is sensitive to response magnitude, each neuron was normalized by its response to its preferred tastant. PCA was applied to mean population responses for each trial type (4 tastants × 2 stimulation conditions), using all baseline and 10-s post-delivery time points. This procedure stabilizes the PCA axes by fitting them to stimulus-averaged responses rather than noisy single-trial responses. Single-trial responses were averaged within the high response–stimulus correlation window (1.5–4.5 s post-delivery) and projected onto the PCA axes derived from trial-type means. Quantifications used the first six principal components (~75% variance).

The palatability axis was defined as the vector connecting palatable (sucrose, NaCl) and aversive (citric acid, quinine) centroids, computed separately for stim and no-stim trials. Responses were projected onto this axis and normalized such that palatable centroid = 1 and aversive centroid = −1. Mixture-ratio and sucrose-concentration axes were defined similarly, except end-range tastants were used. Dynamic range was the difference between projection scores of the end-range tastants. The angle between stim- and no-stim-derived axes were computed using their dot product. LC-induced changes in tastant-pair separation were normalized by the no-stim dynamic range. We used normalized distance differences rather than distance ratios because ratios can become unstable when baseline distances are small.

Dynamic range changes, axis rotations, and pairwise distance changes were assessed by permuting trial labels between stim and no-stim condtions 1,000 times. Permutation-derived null distributions yielded two-tailed *p*-values for dynamic range ratios and one-tailed *p*-values for axis rotation. The latter choice assumed that LC-induced rotation would increase, rather than decrease, the angle that arose from variability in axis estimation. Pairwise distance changes were Bonferroni corrected for six comparisons. Dispersion orthogonal to the attribute axis was calculated as the mean perpendicular distance of points from the axis; LC-induced changes were assessed using the same permutation procedure. Procrustes transformations were used to align centroids between stim and no-stim conditions for visualization in the first three PCs.

For palatability, responses from both trial types were projected onto the no-stim-derived axis, and scores were compared using RM two-way ANOVA (tastant × stim condition) with Bonferroni-corrected *post hoc* tests (performed in GraphPad Prism).

#### Modulation type analysis

Neuronal tuning was defined as mean responses to each tastant during 1.5–4.5 s post-delivery (same window as PCA). For stimulus-discriminative neurons, stim vs. no-stim responses were compared using three models: (i) *Null*: main effect of tastant only; (ii) *Scale–shift*: stimulation modifies tastant tuning via multiplicative and additive effects:

*r*_*stim*_ = *mr*_*no*-*stim*_+ *a*, where *r* denotes the tastant-evoked response, and *m* and *a* represent the multiplicative and additive factors, respectively; (iii) *Flexible*: tastant-specific changes are allowed by including interaction terms between tastant identity and stimulation condition, enabling arbitrary reshaping of the tuning profile. Null and flexible models were fit with linear least squares regression (MATLAB *fitlm* function); scale–shift was fit with nonlinear least squares (*fitnlm*). Models were compared using Akaike Information Criterion (AIC) with hierarchical selection: scale–shift had to outperform null (ΔAIC > 2) to indicate modulation; flexible was selected only if it outperformed both. To prevent unstable nonlinear fits, scale–shift fits were constrained (m: 0–10; a: −10–10). Fits exceeding these bounds typically yielded extreme values and were thus excluded from the scale–shift category and evaluated only against the null and flexible models.

Proportions of scale–shift vs. flexible neurons were compared across experiments using logistic regression (binary response variable: modulation type; predictor: attribute), with pairwise Wald tests and Tukey correction (performed in R).

To relate multiplicative modulation to neuronal contributions to stimulus coding, we quantified each neuron’s contribution to attribute axes from the no-stim condition. Attribute axes, defined in PCA space, were projected back into neural activity space by multiplying each axis vector with the PCA coefficient matrix, yielding a vector of neuronal weights. Each neuron’s contribution was defined as the absolute value of its weight. For scale–shift modulated neurons, multiplicative factors were log-transformed to symmetrize expansive and compressive effects, and their relationship to neuronal contributions was assessed using Pearson’s correlation.

#### Fiber photometry data analysis

Raw photometry signals were preprocessed using a custom R script to linearly unmix the overlapping GRAB_NE2h_ and tdTomato signals, following established protocols.^44,116^ To mitigate artifacts in GRAB_NE2h_ fluorescence signal (e.g., due to tissue displacement, cable bending during animal movement), the ratio of unmixed GRAB_NE2h_ to tdTomato signals (fluorescence ratio) was computed and used for all subsequent analyses, as performed previously.^42,44,45^

Fluorescence ratio traces were analyzed using custom MATLAB scripts. The percent change in fluorescence (%ΔF/F) was calculated by normalizing fluorescence ratios to their baseline mean values.

#### Brief-access taste test analysis

Licking data from the brief-access test were first processed in MATLAB to calculate the mean lick counts for each 10-s trial. Lick counts were then analyzed using an RM one-way ANOVA, followed by Šídák’s post hoc comparisons (performed in GraphPad Prism).

## Supplementary Material

1

SUPPLEMENTAL INFORMATION

Supplemental information can be found online at https://doi.org/10.1016/j.celrep.2026.117990.

## Figures and Tables

**Figure 1.**
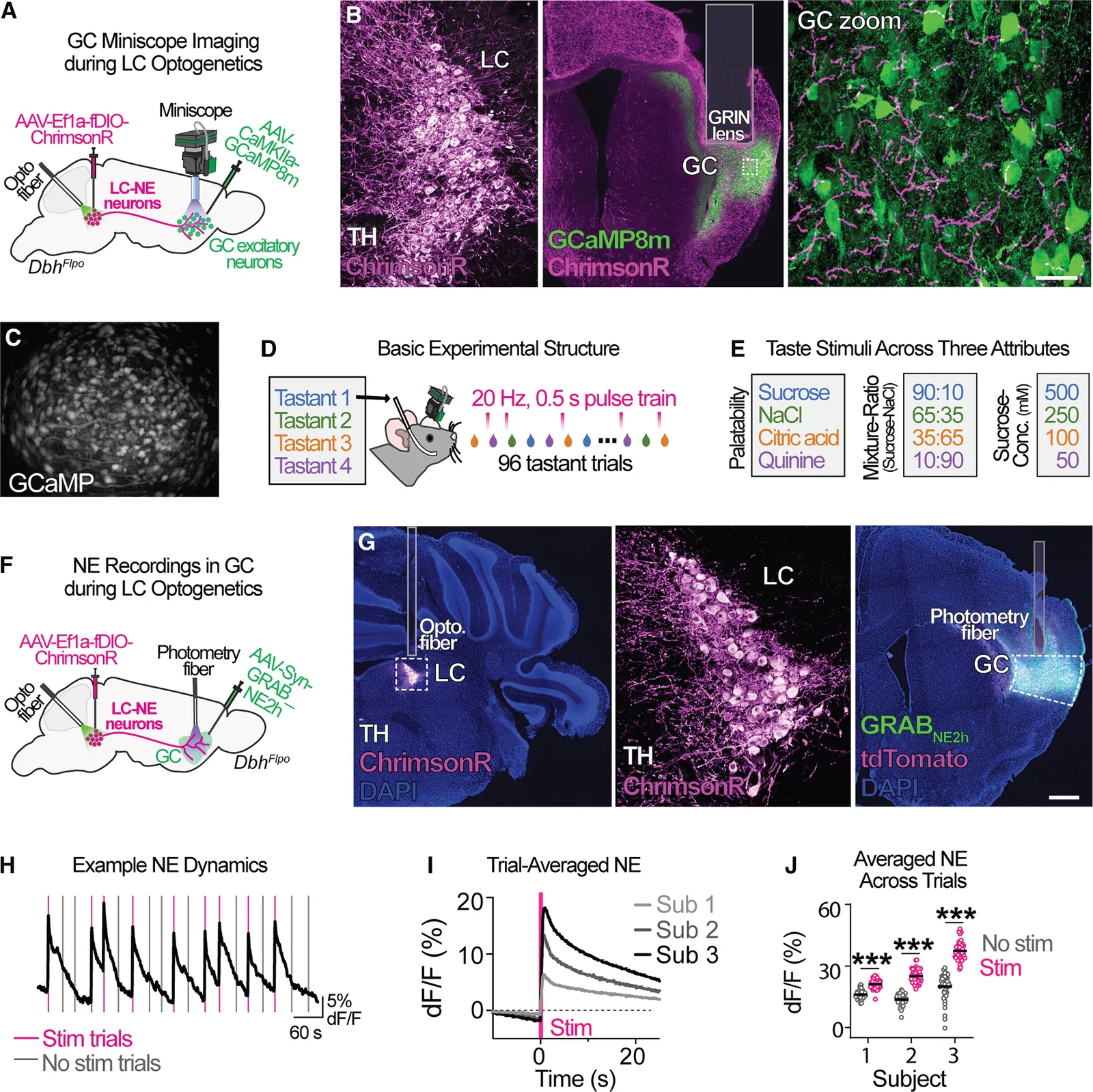
Approaches for assessing phasic LC modulation of taste-evoked GC responses (A) Schematic of the viral-genetic approach and GC calcium imaging during LC optogenetic stimulation. (B) *Left*: ChrimsonR-expressing LC neurons. *Middle*: ChrimsonR-positive LC axons project broadly, including to GC (dashed outline), where the GRIN lens is positioned (gray outline). *Right:* zoomed view of GC showing excitatory neurons expressing GCaMP8m. Scale bars: 101.7 μm (left), 789 μm (middle), and 64.4 μm (right). (C) Example calcium-imaging field of view. (D) *Experimental design*. Mice received one of four pseudo-randomly delivered taste stimuli via IOC. LC neurons were phasically stimulated before taste delivery on half the trials. (E) Taste stimuli used to assess three taste attributes. (F) Schematic of the viral-genetic strategy and NE fiber photometry during LC optogenetic stimulation. (G) *Left*: ChrimsonR-expressing LC neurons; gray line marks optical-fiber placement. *Middle*: higher-magnification view of ChrimsonR-expressing LC neurons. *Right*: GC GRAB_NE2h_ and tdTomato expression; gray line marks optical-fiber placement above GC (dashed outline). Scale bars: 498 μm (left), 66.7 μm (middle), 500 μm (right). (H) Example fiber-photometry trace showing NE dynamics evoked by phasic LC stimulation (20 Hz, 0.5 s); vertical lines denote the example timing of tastant delivery (no tastant was delivered in this validation experiment). (I) Trial-averaged NE fluorescence aligned to stimulation onset. *n* = 3 mice. (J) NE fluorescence averaged over 1-s epochs for phasic-stimulation and no-stimulation conditions across 3 mice. Dots indicate mean responses for *n* = 48 trials/condition; horizontal lines indicate condition means. two-way ANOVA: stimulation, *F*_2,282_ = 648.6, *p* < 0.0001; stimulation × subjects, *F*_2,282_ = 63.19, *p* < 0.0001; subjects, *F*_2,282_ = 210.7, *p* < 0.0001. Šídák’s post-hoc test: ***p < 0.001, stim vs. no stim.

**Figure 2.**
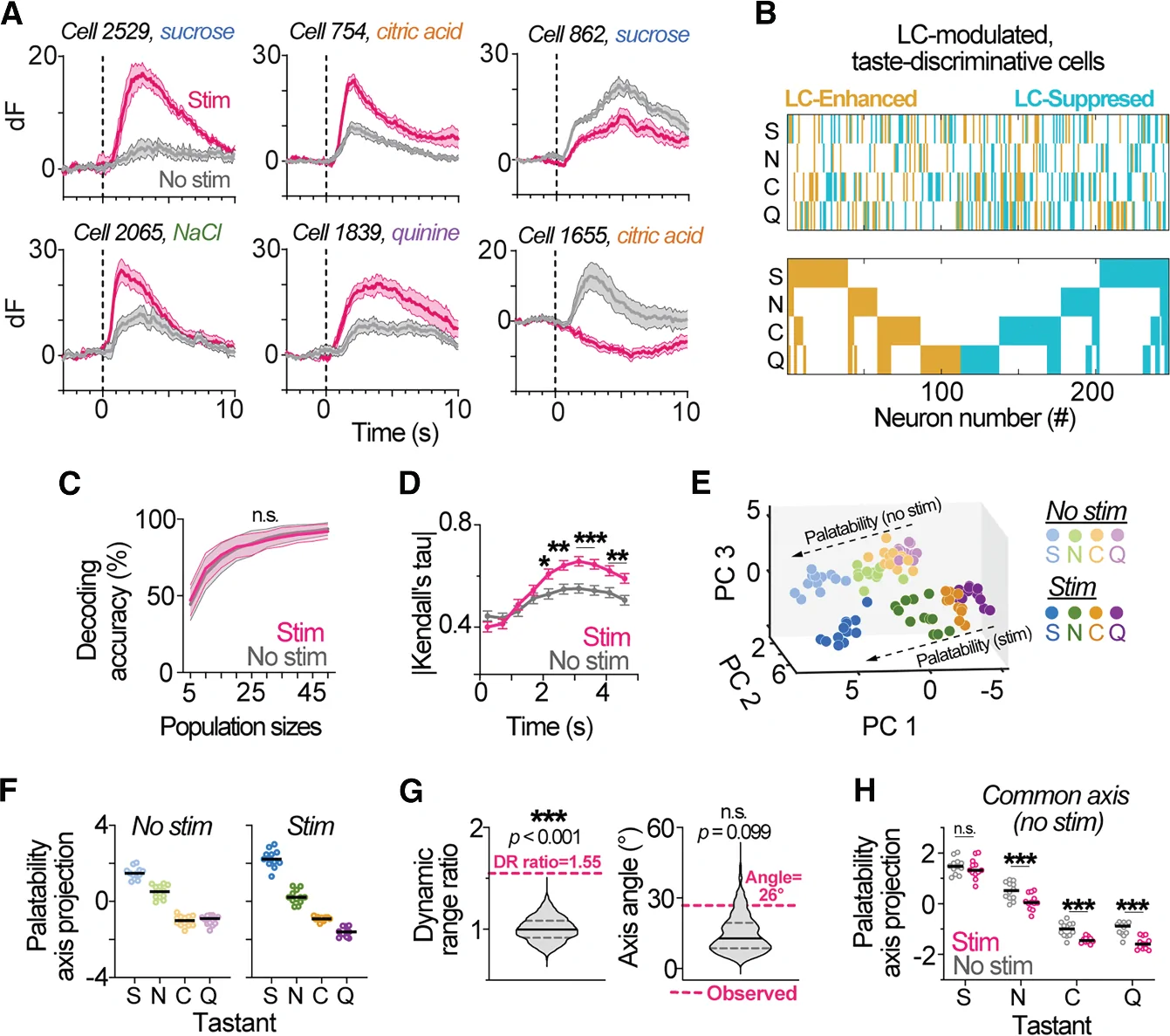
Phasic LC activation bidirectionally modulates taste-evoked responses and reorganizes GC palatability representation (A) Mean taste-evoked responses from six representative GC neurons with and without LC stimulation. Tastants were delivered at 0 s; shading indicates ±SEM. (B) *Top*: modulation direction of all LC-modulated GC neurons. Each tick shows the effect of LC activation on responses to sucrose (S), NaCl (N), citric acid (C), or quinine (Q). Gold and teal indicate LC-enhanced and LC-suppressed cell-taste pairs, respectively. *Bottom*: same data sorted by modulation direction. (C) Decoding accuracy for LC-modulated, taste-discriminative GC neurons across population sizes. Data are mean ± SD of bootstrapped means. Non-significant (n.s.) permutation tests. (D) Correlations between response magnitude and palatability rank across LC-modulated neurons and time bins. Two-way RM ANOVA: time × stimulation (*F*_5.766, 1418_= 6.259, *p* < 0.0001); stimulation (*F*_1.000, 246.0_= 13.41, *p* = 0.0001); time (*F*_5.112, 1258_= 33.98, *p* < 0.0001). Bonferroni post hoc: ***p < 0.001, **p < 0.01, *p < 0.05. Mean ± SEM (247 cells). (E) PCA of LC-modulated GC population responses to tastants (12 trials/condition). Arrows indicate palatability axes. (F) Projection of responses onto the corresponding palatability axis. (G) Permutation tests for dynamic-range ratio (left) and palatability-axis angle (right) in LC-modulated neurons. Violin plots show null distributions (median, solid line; quartiles, dashed); magenta lines indicate observed values. (H) Responses projected onto a common palatability axis defined from no-stimulation trials. two-way ANOVA: tastant (*F*_3,88_= 482.7, *p* < 0.0001); stimulation (*F*_1,88_= 40.22, *p* < 0.0001); tastant × stimulation (*F*_3,88_= 2.824, *p* = 0.0433). Bonferroni’s post hoc: ***p < 0.001 Black lines indicate means; dots represent individual trials (12 trials/condition).

**Figure 3.**
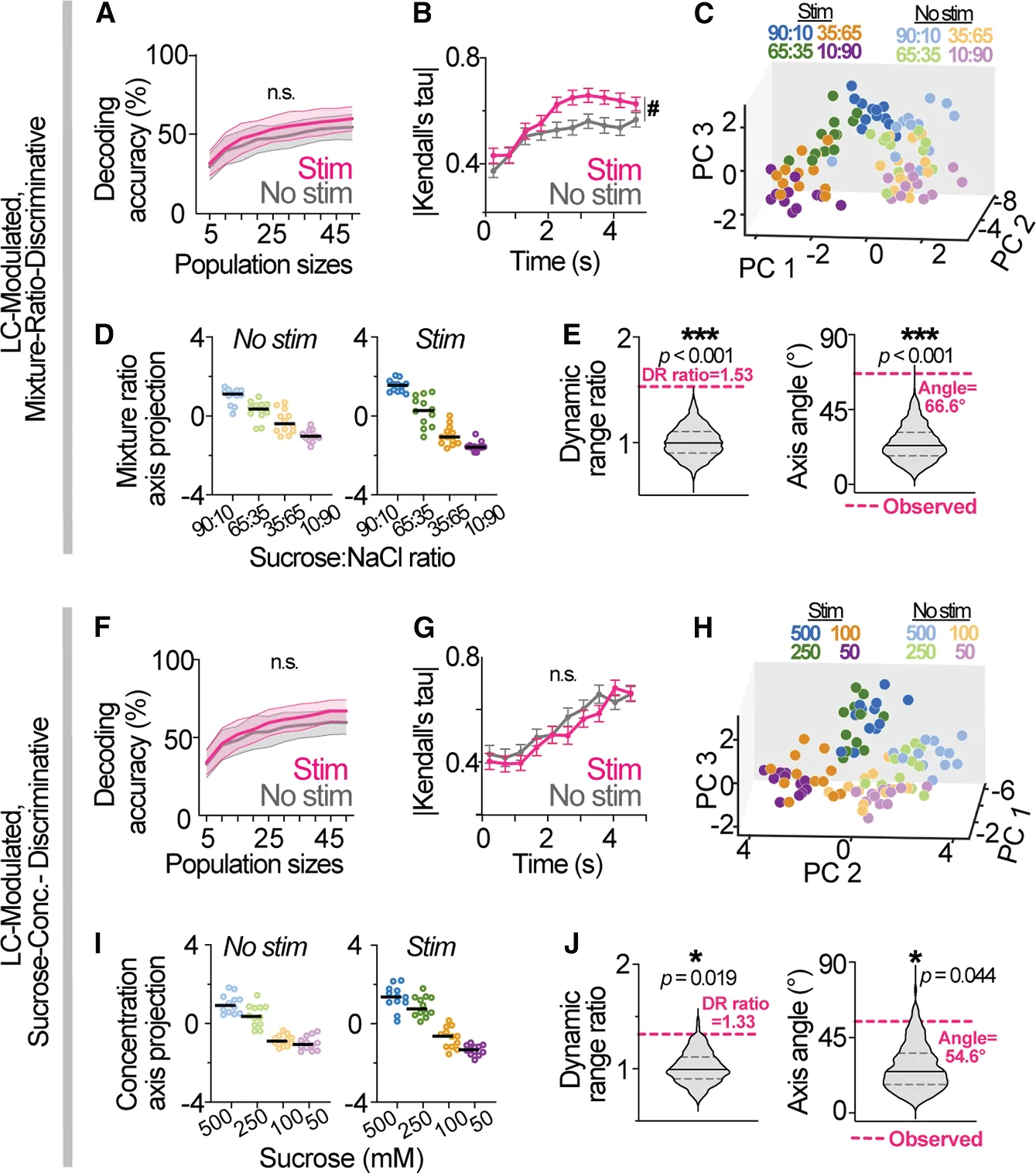
Phasic LC activation reshapes GC representations of mixture composition and sucrose intensity (A–E) Mixture-ratio experiment in LC-modulated neurons. (A) Decoding accuracy across population sizes. Values are mean ± SD of bootstrapped means; non-significant (n.s.) permutation tests. (B) Correlations between response magnitude and mixture-ratio rank across neurons and time bins. Two-way RM ANOVA: stimulation (*F*_1.000, 122.0_= 6.169, *p* = 0.0144); time *F_5.565, 679_= 20.79, *p* < 0.0001); time × stimulation (*F*_5.128, 625.7_= 1.597, *p* = 0.1570). ^#^*p* < 0.05, main effect of stimulation. Mean ± SEM (123 cells). (C) Population responses projected into PCA space (12 trials/condition). Arrows indicate mixture-ratio axes. (D) Projection onto the corresponding mixture-ratio axis. (E) Permutation tests for dynamic-range ratio (left) and axis angle (right). (F–J) Sucrose-concentration experiment in LC-modulated neurons. (F) Decoding accuracy across population sizes. Values are mean ± SD of bootstrapped means. (G) Correlations between response magnitude and sucrose-concentration rank across neurons and time bins. Two-way RM ANOVA: stimulation (*F*_1.000, 98.0_= 0.7657, *p* = 0.3837); time (*F*_4.870, 477.2_= 26.38, *p* < 0.0001); time × stimulation (*F*_5.594, 548.3_= 1.111, *p* = 0.3543). Mean ± SEM (99 cells). (H) Population responses projected into PCA space (12 trials/condition). Arrows indicate sucrose-concentration axes. (I) Projection onto the corresponding sucrose-concentration axis. (J) Permutation tests for DR ratio (left) and concentration-axis angle (right).

**Figure 4.**
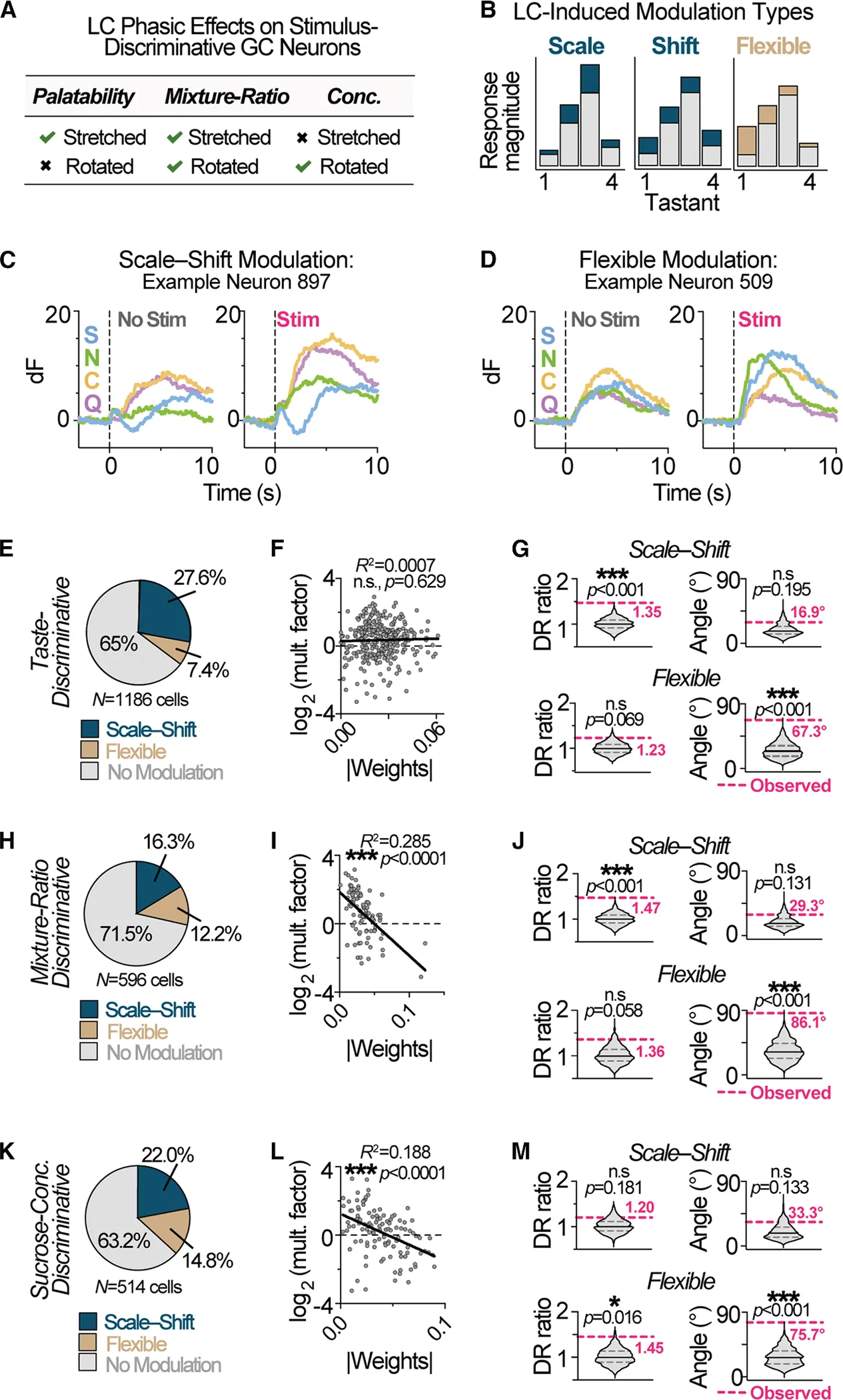
Phasic LC activation reshapes neuronal tuning in an attribute-dependent manner (A) Summary of LC-induced transformations across stimulus-discriminative GC neurons. (B) Schematic of scale, shift, and flexible modulation. Gray indicates tuning without LC stimulation. (C and D) Example GC neurons showing scale-shift (C) or flexible modulation (D) during no-stimulation (left) and stimulation (right) trials. Curves show mean responses to sucrose (S), NaCl (N), citric acid (C), and quinine (Q). (E, H, and K) Proportion of modulation types among basic taste (E), mixture ratio (H), and sucrose-concentration discriminative (K) GC neurons. (F, I, and L) Linear regressions of absolute modulation weight versus log_2._

**Figure 5.**
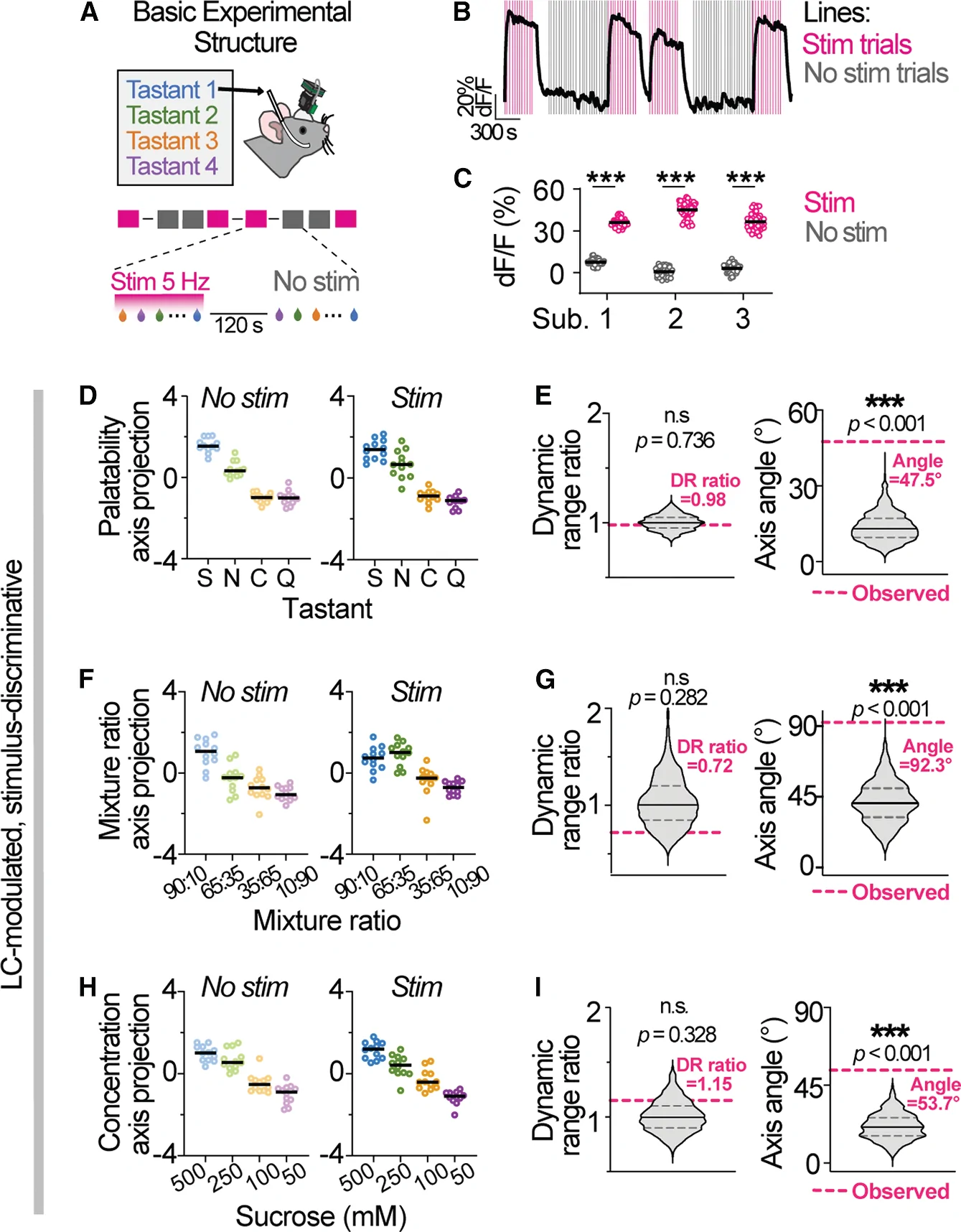
Effects of prolonged tonic LC activation on GC representation of taste attributes (A) Tastant deliveries were divided into eight blocks (12 deliveries per block). Tonic LC photostimulation (5 Hz) occurred in four blocks. Each stimulation block is followed by a gap period (145–155 s) before the onset of the next block. (B) Example fiber-photometry trace showing NE dynamics evoked by tonic LC stimulation; vertical lines indicate tastant timing (no tastant was delivered in this validation experiment). (C) NE fluorescence averaged over 1-s epochs for tonic-stimulation and no-stimulation trials. two-way ANOVA: stimulation (*F*_2,282_ = 5392, *p* < 0.0001); stimulation × subjects (*F*_2,282_ = 97.60, *p* < 0.0001); subjects (*F*_2,282_ = 14.00, *p* < 0.0001). Šídák’s post hoc: ***p <0.001. Dots show *n* = 48 trials/condition (3 mice); lines show means. (D, F, and H) Population responses of LC-modulated neurons projected onto the palatability (D), mixture-ratio (F) and sucrose-concentration (H) axes for stimulation (right) and no-stimulation (left) trials (12 trials/condition). (E, G, and I) Permutation tests for dynamic-range ratios (left) and axis angles (right) for tonic LC-modulated neurons in the palatability (E), mixture-ratio (G), and sucrose-concentration (I) experiments. Violin plots show permuted distributions (median, solid black; quartiles, dashed), with observed values in magenta.

**KEY RESOURCES TABLE:** 

REAGENT or RESOURCE	SOURCE	IDENTIFIER

Antibodies		

Anti-GFP (chicken)	Abcam	Abcam Cat # ab13970; RRID: AB_300798
Anti-dsRed (rabbit)	Takara	Takara Cat # 632496; RRID: AB_10013483
Anti-TH (mouse)	GeneTex	GeneTex Cat # GTX10372; RRID: AB_373127
Goat anti-Chicken Alexa Fluor 488	Thermo Fisher Scientific	Thermo Fisher Scientific Cat # A-11039; RRID: AB_2534096
Goat anti-Rabbit Alexa Fluor 568	Thermo Fisher Scientific	Thermo Fisher Scientific Cat # A-11036; RRID: AB_10563566
Goat anti-Mouse Alexa Fluor 633	Thermo Fisher Scientific	Thermo Fisher Scientific Cat # A-21052; RRID: AB_2535719

Bacterial and Virus Strains		

AAV-Ef1a-fDIO-ChrimsonR-tdTomato	Gift from Patricia Jensen	Addgene plasmid # 171027; RRID: Addgene_171027; Viral prep: NIEHS Viral Vector Core
AAV-Ef1a-fDIO-tdTomato	Gift from Patricia Jensen	Addgene plasmid # 128434; RRID: Addgene_128434; Viral prep: NIEHS Viral Vector Core
AAV-CaMKIIa-jGCaMP8m-WPRE	Gift from Loren Looger	Addgene viral prep # 176751-AAV9; RRID: Addgene_176751
AAV-hSyn-GRAB-NE2h	Gift from Yulong Li	WZ Biosciences; viral prep # L003011-AAV9
AAV-CAG-tdTomato	Gift from Edward Boyden	Addgene viral prep # 59462-AAV9; RRID: Addgene_59462

Deposited Data		

Dataset	This paper	Zenodo: doi:10.5281/zenodo.21826182
Analysis code	This paper	Zenodo: doi:10.5281/zenodo.21826182

Experimental Models: Organisms/Strains		

Mouse; *Dbh*^Flpo^	The Jackson Laboratory	JAX Stock # 033952; RRID: IMSR_JAX:033952

Software and Algorithms		

Fiji (ImageJ2)	NIH	https://imagej.net/software/imagej2/; RRID: SCR_002285
Illustrator	Adobe	https://www.adobe.com/products/illustrator.html; RRID: SCR_010279
Inscopix Data Acquisition Software (IDAS)	Inscopix	https://inscopix.com/software-analysis-miniscope-imaging/
Inscopix Data Processing Software (IDPS)	Inscopix	https://inscopix.com/software-analysis-miniscope-imaging/
Leica Application Suite X (LASX)	Leica Microsystems	https://www.leica-microsystems.com/products/microscope-software/p/leica-las-x-ls/; RRID: SCR_013673
MATLAB	The MathWorks	https://www.mathworks.com/products/matlab.html; RRID: SCR_001622
OceanView 2.0	OceanOptics	https://www.oceanoptics.com/software/oceanview/
Prism 10	GraphPad	https://www.graphpad.com/; RRID: SCR_002798

Other		

nVoke 2.0 miniscope system	Inscopix	https://inscopix.com/nvoke-system/; RRID: SCR_023028
Coherent OBIS 488 nM Laser System, 20 mW	Edmund Optics	https://www.edmundoptics.com/p/obis-488nm-ls-20mw-laser/29838/; Edmund Optics Cat #: 87–459
Green 532 nM DPSS Laser	Shanghai Laser & Optics Century Co.	https://www.lasercentury.com/product.asp?id=609; Shanghai Laser Cat #: GL532T3–200FC
MedPC Behavioral Control	Med Associates Inc.	https://med-associates.com/product/med-pc/; RRID: SCR_012156
OCEAN FX Miniature Spectrometer	Ocean Optics	https://www.oceanoptics.com/spectrometer/ocean-fx/; Ocean Optics Cat #: OCEAN-FX-VIS-NIR-ES
VC-Plus-4 Channel Gravity System	ALA Scientific Instruments	https://alascience.com/products/vcplus-4-channel-gravity-systems/; ALA Cat #: ALAVCPLUS-4CLG

## Data Availability

For questions or additional information, please contact the lead contact, Natale R. Sciolino (natale.sciolino@uconn.edu). All reasonable requests will be accommodated. No new or unique reagents were generated. Data: All neural and behavioral datasets generated here have been deposited at Zenodo (doi:10.5281/zenodo.21826182). Code: Original analysis code used has been deposited at Zenodo (doi:10.5281/zenodo.21826182). Other items: Any additional information required to reanalyze the data reported in this paper is available from the lead contact upon request.

## Highlights

- Phasic locus coeruleus (LC) activation reorganizes gustatory cortex coding
- LC activation expands representational distance along a palatability axis
- Gain modulation and flexible tuning changes underlie LC-induced population effects
- Tonic LC activation produces limited effects on gustatory coding
