# Supplementary material for: Locus coeruleus activation transforms cortical taste representations
Source: Cell Rep. Author manuscript; Available in PMC 2026 Oct 14. (PMC13663908; doi:10.1016/j.celrep.2026.117990)
Supplement: Supplementary file 1 [file NIHMS2212249-supplement-1.pdf]

Cell Reports, Volume *45*

Supplemental information

Locus coeruleus activation transforms cortical taste representations

Will Fan, Paula To, and Natale R. Sciolino

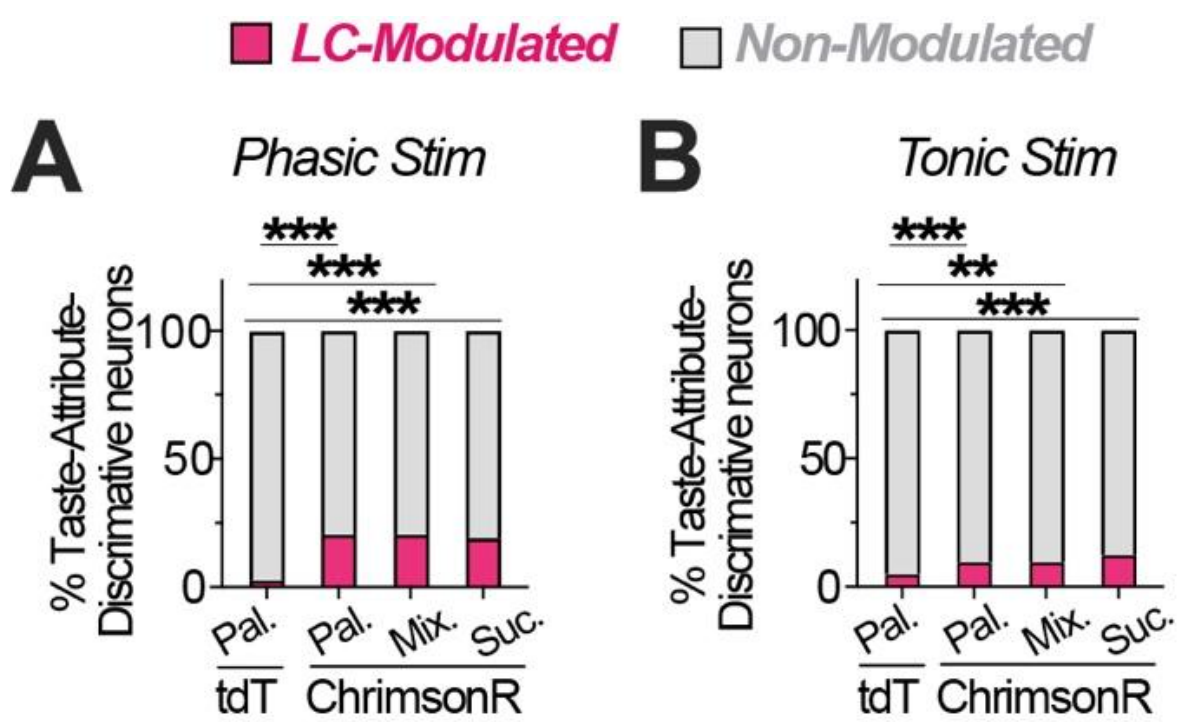

Figure S1. ChrimsonR Mice Have More GC neurons Identified as Modulated versus tdTomato Control.

(A) Proportion of GC neurons identified as LC-modulated during phasic stimulation in tdTomato (tdT) control and ChrimsonR groups for palatability (Pal.), mixture ratio (Mix.), and sucrose concentration (Suc.) experiments. Across all attributes, ChrimsonR mice have more neurons modulated than tdT control (exact binomial tests): Palatability, 20.83% vs. 2.88% (****P*<0.0001, 95% CI: 18.55–23.25%); Mixture ratio, 20.64% vs. 2.88% (****P*<0.0001, 95% CI: 17.46–24.11%); Sucrose concentration, 19.26% vs. 2.88% (****P*<0.0001, 95% CI: 15.94–22.94%).

(B) Proportion of GC neurons classified as LC-modulated during tonic stimulation across taste attributes. ChrimsonR mice have more neurons modulated than tdT control (exact binomial tests): Palatability, 9.66% vs. 5.02% (****P*<0.0001, 95% CI: 7.87–11.70%); Mixture ratio, 9.85% vs. 5.02% (***P*<0.01, 95% CI: 6.59–14.01%); Sucrose concentration, 10.14% vs. 5.02% (****P*<0.0001, 95% CI: 7.66–13.08%).

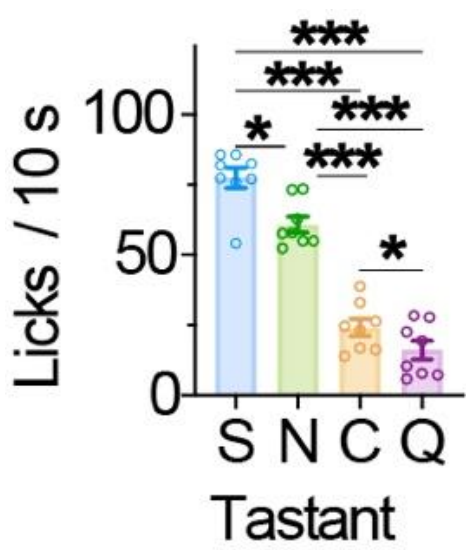

Figure S2. Licking Responses to Four Basic Tastes in the Brief-Access Test.
Mean lick counts during 10-s, brief access to 200 mM sucrose (S), 50 mM salt (N), 20 mM citric acid (C), and 1 mM quinine (Q). A one-way RM ANOVA showed a significant tastant effect, $F_{1.538,10.76}$= 126.7, P<0.0001. Šídák's post-hoc tests confirmed the palatability order S > N > C > Q (***P<0.001, *P<0.05). Data are mean ± SEM; dots represent individual mice (n=8).

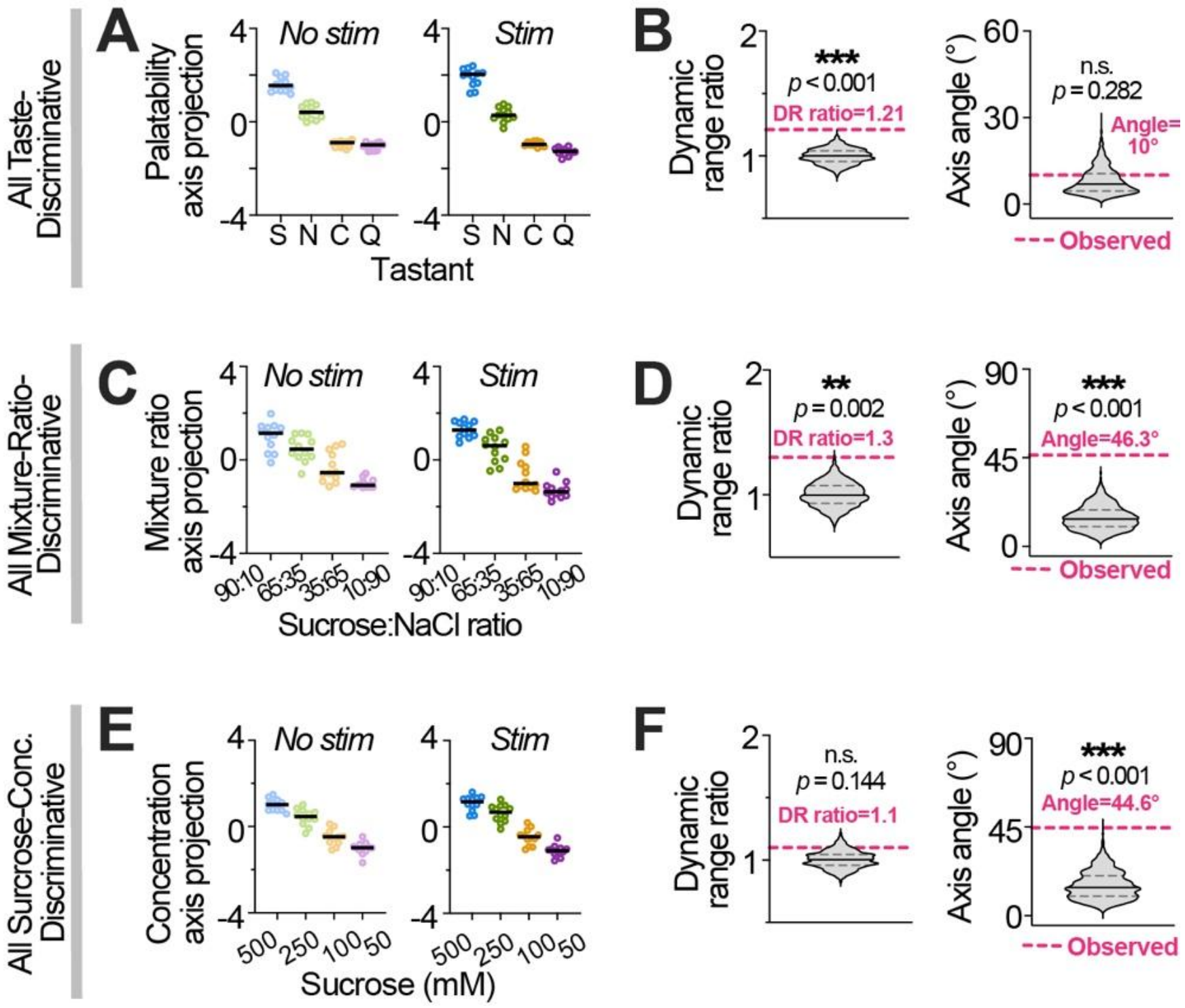

Figure S3. Effects of Phasic LC Activation on Stimulus Representation in All Stimulus-Discriminative Neurons.

(A–B) All taste-discriminative neurons. (A) Population responses projected onto the corresponding palatability axis for stimulation (right) and no-stimulation (left) trials (12 trials/condition). (B) Permutation tests for dynamic-range (DR) ratio (left) and palatability-axis angle (right).

(C–D) All mixture-ratio-discriminative neurons. (C) Population responses projected onto the corresponding mixture-ratio axis for stimulation (right) and no-stimulation (left) trials (12 trials/condition). (D) Permutation tests for DR ratio (left) and mixture-ratio-axis angle (right).

(E–F) All sucrose-concentration-discriminative neurons. (E) Population responses projected onto the corresponding concentration axis for stimulation (right) and no-stimulation (left) trials (12 trials/condition). (F) Permutation tests for DR ratio (left) and concentration-axis angle (right).

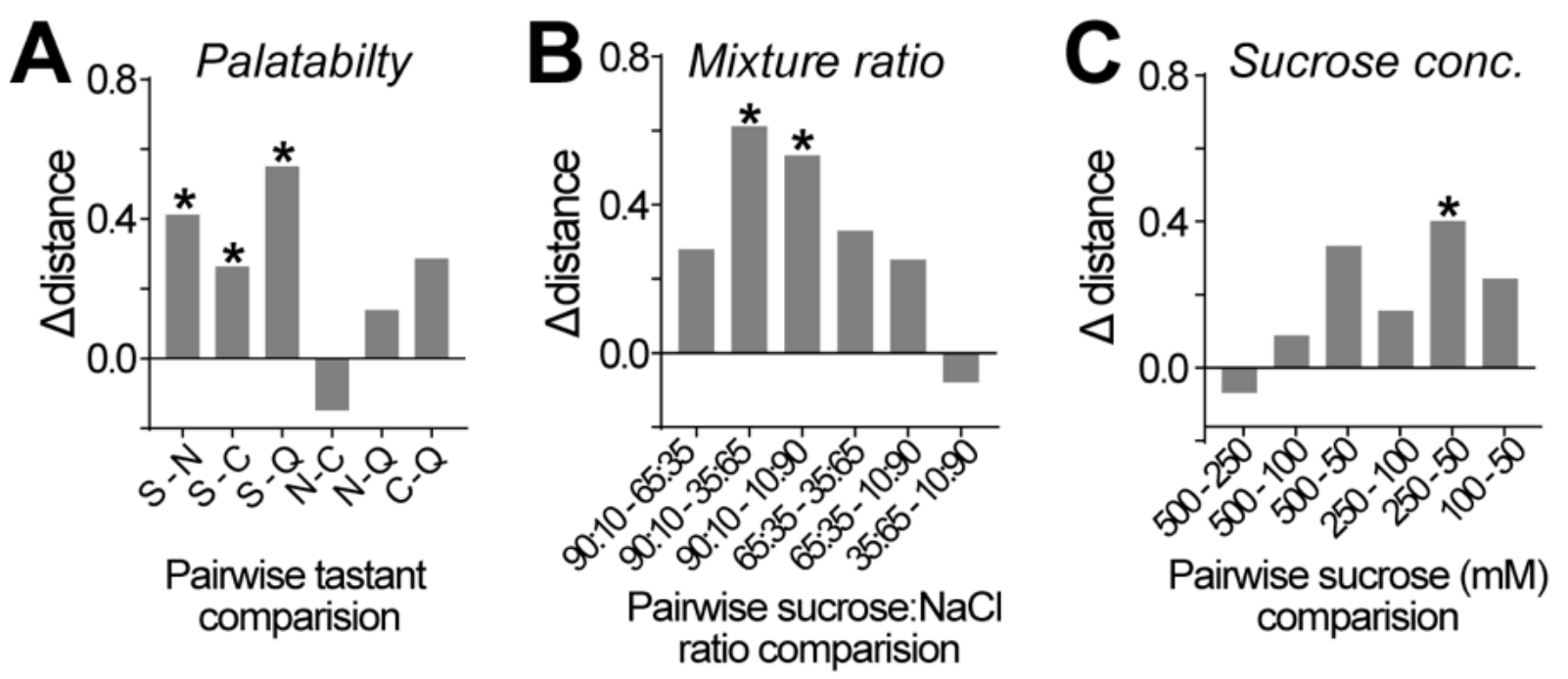

Figure S4. Phasic LC Activation Non-uniformly Alters Tastant Separation Along Attribute-Relevant Axes.
Changes in pairwise centroid separation (Δ distance) for (A) palatability, (B) mixture ratio, and (C) sucrose concentration. Δ distance was normalized to the no-stim dynamic range, with positive values indicating increased separation during phasic LC activation. Asterisks indicate significant permutation-test comparisons after Bonferroni correction ($p < 0.05$).

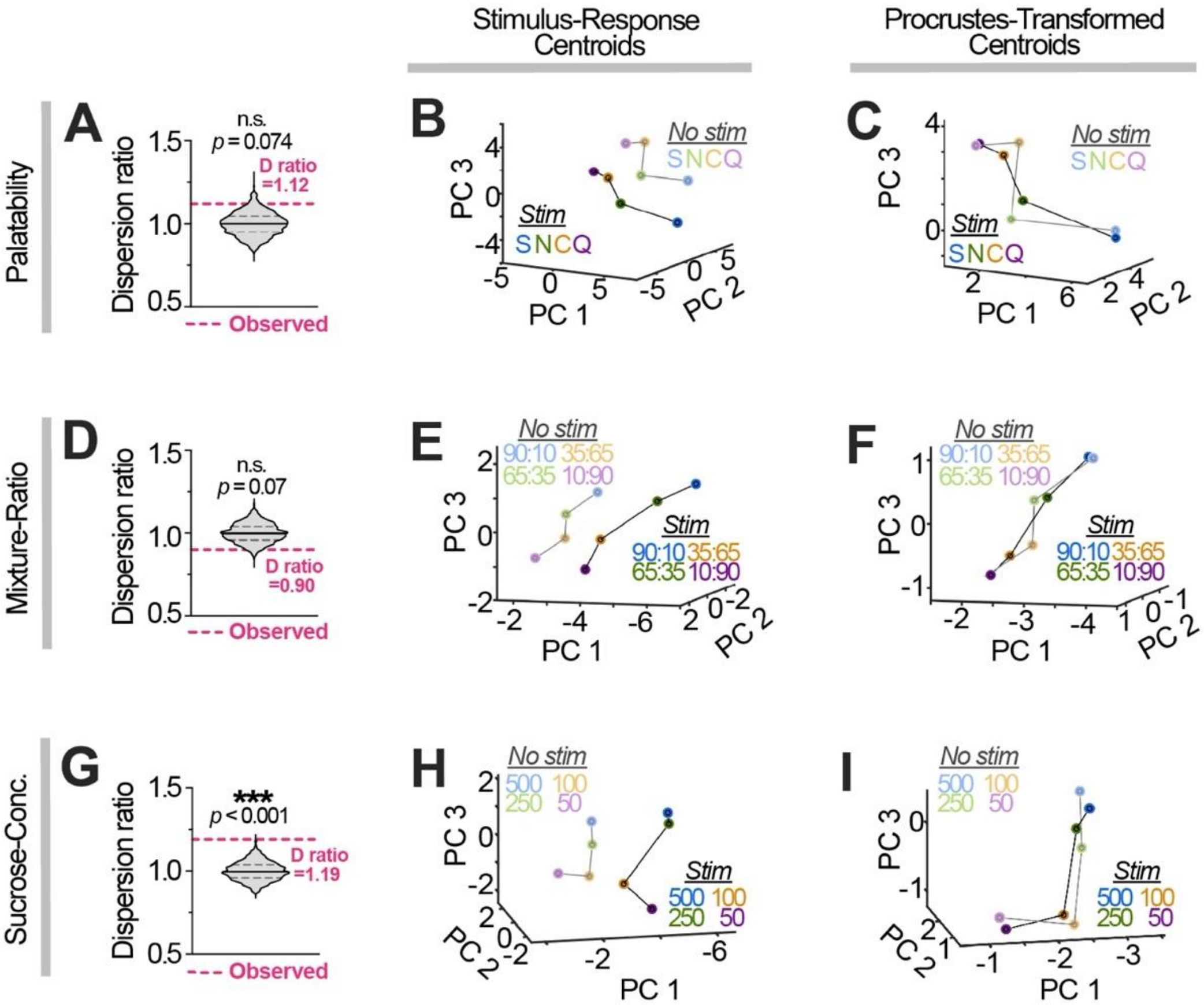

Figure S5. Effects of Phasic LC Activation on Tastant Responses in Directions Orthogonal to the Coding Axes in LC-Modulated GC Neurons.

(A, D, G) Permutation tests for dispersion ratios in phasic LC-modulated GC neurons in the palatability (A), mixture-ratio (D), and sucrose-concentration (G) experiments. Dispersion is defined as the mean vertical distance of each taste response from the coding axis. The dispersion ratio compares stimulation vs. no-stimulation conditions. Violin plots show permuted distributions (median, solid black; quartiles, dashed), with observed ratios in magenta.

(B, E, H) Stimulus-response centroids for LC-modulated GC neurons in the palatability (B), mixture-ratio (E), and sucrose-concentration (H) experiments, projected into principal-component space with and without stimulation. Stimuli are as follows: palatability—sucrose (S), NaCl (N), citric acid (C), and quinine (Q); mixture-ratio—sucrose:salt ratios; sucrose-concentration—listed concentrations (mM).

(C, F, I) Stimulation centroids aligned to the no-stimulation centroids using Procrustes analysis for LC-modulated GC neurons in the palatability (C), mixture-ratio (F), and sucrose-concentration (I) experiments. Stimuli are as listed above.

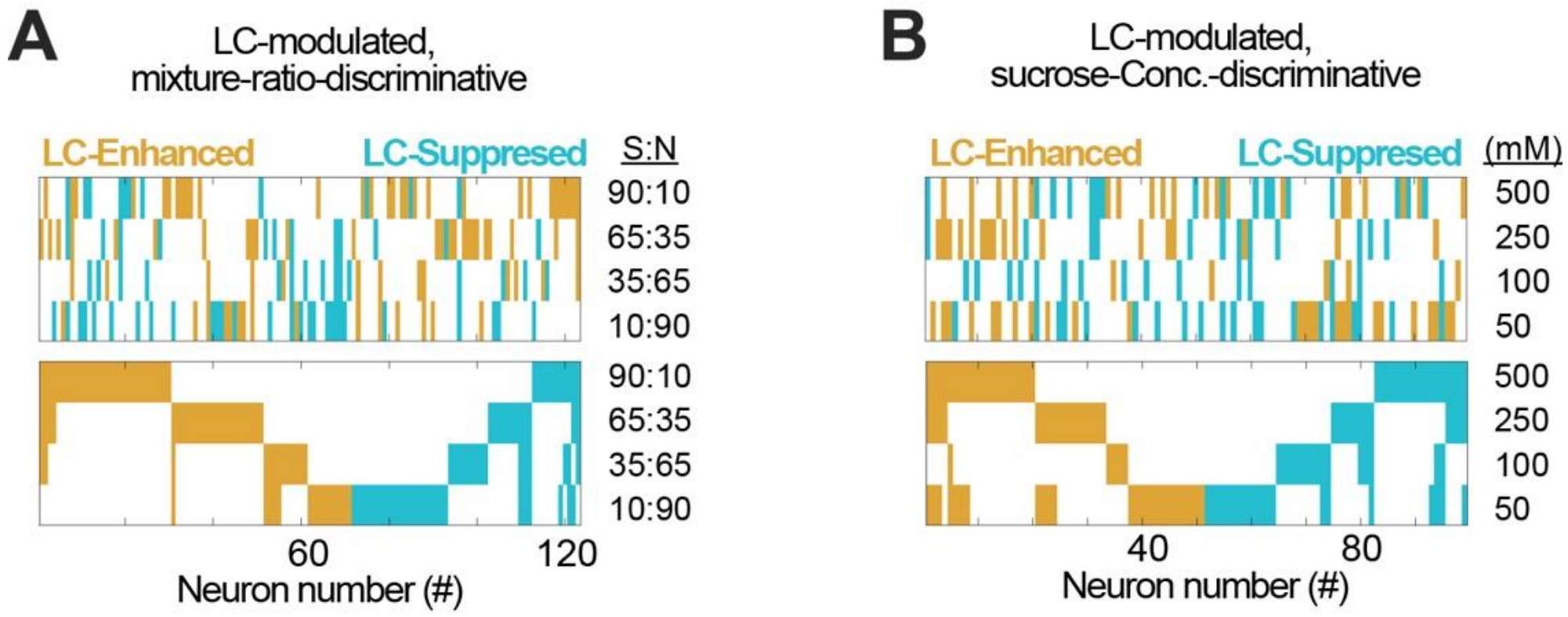

Figure S6. Effects of Phasic LC Activation on Tastant Responses in Directions Orthogonal to the Coding Axes in LC-Modulated GC Neurons.

(A) *Top*: Modulation direction for LC-modulated GC neurons. Each tick shows the effect of LC activation on responses to sucrose (S)–NaCl (N) mixture ratios; colors indicate modulation direction. *Bottom*: Same data sorted by modulation direction.

(B) *Top*: Modulation direction for LC-modulated GC neurons. Each tick shows the effect of LC activation on responses to different sucrose (S)–NaCl (N) mixture ratios; colors indicate modulation direction. *Bottom*: Same data sorted by modulation direction.

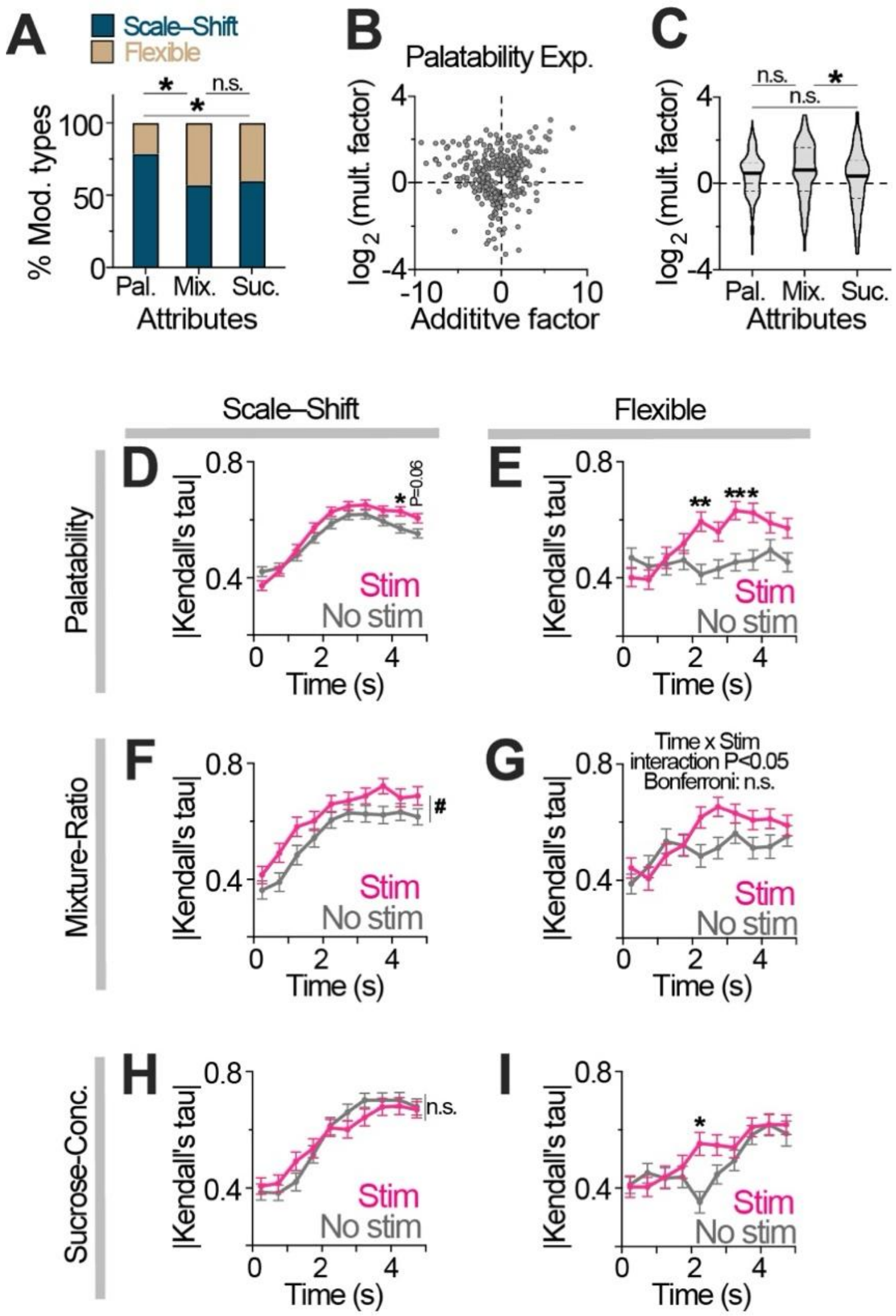

Figure S7. Additional Analysis of Scale–Shift and Flexible Modulation during Phasic LC Stimulation.

(A) Proportion of GC neurons showing scale–shift or flexible modulation during phasic LC stimulation in the palatability (Pal.), mixture ratio (Mix.), and sucrose concentration (Suc.) experiments. Logistic regression revealed a significant effect of stimulus condition on the relative prevalence of scale-shift versus unconstrained modulation (likelihood-ratio test: $\chi^2_{df=2}$= 37.80, P=6.21 × 10^{-9}). Tukey-adjusted Wald tests showed that palatability stimuli had significantly higher odds of scale-shift modulation than both mixture (odds ratio = 2.8, P<0.0001) and sucrose stimuli (odds ratio = 2.5, P<0.0001), whereas mixture and sucrose conditions did not differ (odds ratio = 0.89, P = 0.86).

(B) Additive factor versus log multiplicative factor for scale–shift-modulated GC neurons in the palatability experiment.

(C) Distribution of log multiplicative factors for scale–shift modulated GC neurons across taste attributes. Kruskal-Wallis: H_3=6.716, P=0.0348. Dunn's post-hoc: *P<0.05. n.s., non-significant.

(D–I) Time-resolved correlations between neuron's response magnitude and rank for palatability (D–E), mixture ratio (F–G), and sucrose concentration (H–I), shown separately for GC neurons exhibiting scale–shift (D, F, H) or flexible (E, G, I) modulation during phasic LC stimulation.

(D–E) Palatability correlations. Scale–shift neurons (D): Two-way RM ANOVA–Time × stimulation: $F_{6.240,\ 2034}$= 3.225, P=0.0032; Time: $F_{4.924,\ 1605}$= 66.54, P<0.0001; Stimulation: $F_{1.000,\ 326.0}$= 4.037, P=0.0453. Flexible neurons (E): Time × stimulation: $F_{4.795,\ 417.2}$= 5.090, P=0.0002; Time: $F_{4.630,\ 402.8}$= 5.053, P=0.0003; Stimulation: $F_{1.000,\ 87.0}$= 7.444, P=0.0077. Bonferroni post-hoc: **P<0.01, *P<0.05. Mean ± SEM (327 scale–shift cells, 88 flexible cells).

(F–G) Mixture-ratio correlations. Scale–shift neurons (F): Two-way RM ANOVA–Time × stimulation: $F_{5.779,\ 554.8}$= 0.4601, P=0.8215; Time: $F_{5.014,\ 481.4}$= 30.27, P<0.0001; Stimulation: $F_{1.000,\ 96.0}$= 6.588, P=0.0118. #P<0.05, stimulation main effect. Flexible neurons (G): Time × stimulation: $F_{5.050,\ 363.6}$= 2.422, P=0.0348; Time: $F_{5.662,\ 407.7}$= 8.859, P<0.0001; Stimulation: $F_{1.000,\ 72.0}$= 2.091, P=0.1525. n.s., non-significant. Mean ± SEM (97 scale–shift cells, 73 flexible cells).

(H–I) Sucrose-concentration correlations. Scale–shift neurons (H): Two-way RM ANOVA–Time × stimulation: $F_{6.081,\ 681.1}$= 1.639, P=0.1325; Time: $F_{4.992,\ 559.1}$= 44.69, P<0.0001; Stimulation: $F_{1.000,\ 112.0}$= 0.01902, P=0.8906. Flexible neurons (I): Time × stimulation: $F_{5.128,\ 384.6}$= 2.719, P=0.0188; Time: $F_{5.204,\ 390.3}$= 13.70, P<0.0001; Stimulation: $F_{1.000,\ 75.0}$= 1.203, P=0.2762. Bonferroni's post-hoc: *P<0.05. Mean ± SEM (113 scale–shift cells, 76 flexible cells).

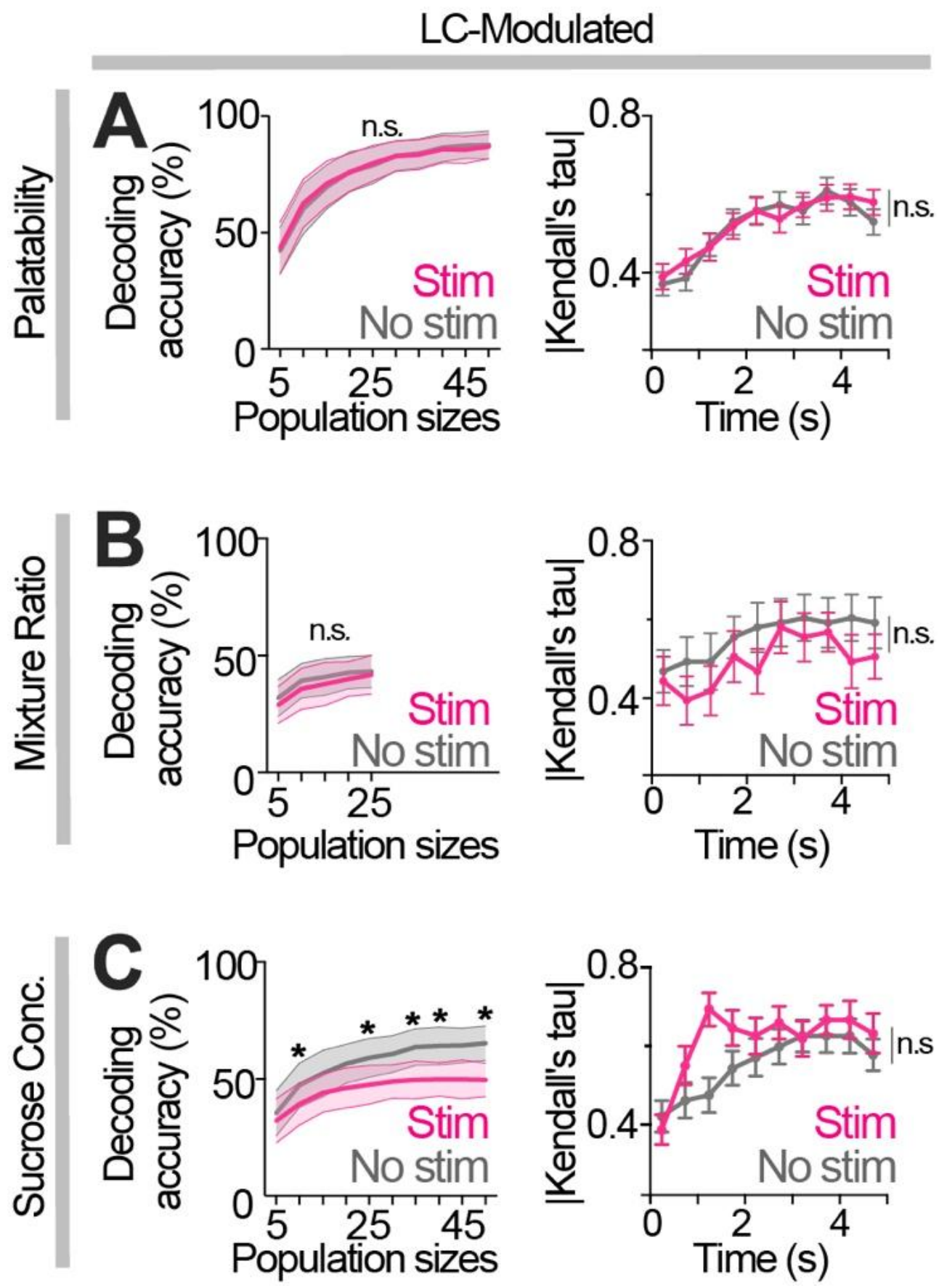

Figure S8. Effects of Prolonged Tonic LC Activation on Population Decoding and Stimuli-Rank Correlations in LC-Modulated GC Neurons.

(A–C) *Left:* Decoding accuracy across population sizes for tonic LC-modulated GC neurons in the palatability (A), mixture-ratio (B), and sucrose-concentration (C) experiments (mean ± SD of bootstrapped means). Permutation tests show no significant effects for palatability and mixture ratio experiments, whereas decoding accuracy was reduced under stimulation in the sucrose-concentration experiment. *P<0.05. *Right*: Time-resolved correlations between each neuron's response magnitude and stimuli rank for each taste attribute. Palatability-discriminative neurons (A): Two-way RM ANOVA–Time × stimulation: $F_{5.072,\ 466.6}$= 0.6119, P=0.6932; Time: $F_{4.769,\ 438.7}$= 17.46, P<0.0001; Stimulation: $F_{1.000,\ 92.00}$= 0.04783, P=0.8274. Mixture-ratio-discriminative neurons (B): Time × stimulation: $F_{3.758,\ 97.71}$= 0.2440, P=0.9033; Time: $F_{4.604,\ 119.7}$= 2.789, P=0.0235; Stimulation: $F_{1.000,\ 26.00}$= 1.322, P=0.2607. Sucrose-concentration-discriminative neurons (C): Time × stimulation: $F_{5.910,\ 283.7}$= 2.135, P=0.0505; Time: $F_{4.112,\ 197.4}$= 7.428, P<0.0001; Stimulation: $F_{1.000,\ 48.00}$= 2.533, P=0.1180. n.s., non-significant stimulation effects. Mean ± SEM (93 palatability cells; 27 mixture-ratio cells; 49 sucrose-concentration cells).

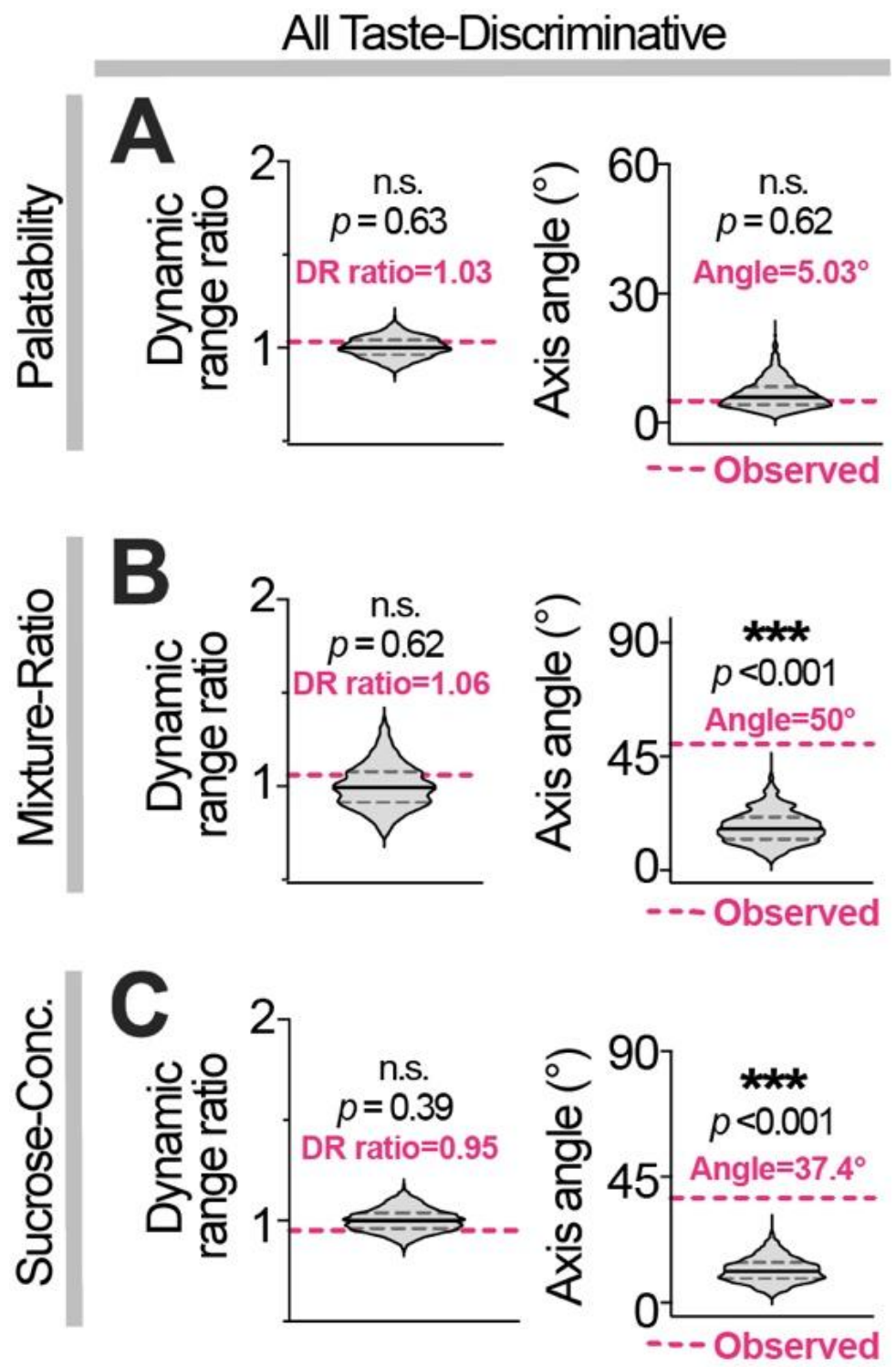

Figure S9. Prolonged Tonic LC Activation Had No Axis-Stretching Effects and Rotated Palatability and Mixture-Ratio Axes.
(A–C) Permutation tests for dynamic-range (DR) ratios (left) and axis angles (right) in all stimulus-discriminative GC neurons during tonic LC stimulation for the palatability (A), mixture ratio (B), and sucrose concentration (C) experiments. Violin plots show permuted distributions (median, solid black; quartiles, dashed), with observed values shown in magenta.
